# 5G Wireless Communication and Health Effects—A Pragmatic Review Based on Available Studies Regarding 6 to 100 GHz

**DOI:** 10.3390/ijerph16183406

**Published:** 2019-09-13

**Authors:** Myrtill Simkó, Mats-Olof Mattsson

**Affiliations:** SciProof International AB, Vaktpoststigen 4, 83132 Östersund, Sweden; mats-olof.mattsson@sciproof-international.se

**Keywords:** radiofrequency electromagnetic fields, MMW, in vivo, in vitro

## Abstract

The introduction of the fifth generation (5G) of wireless communication will increase the number of high-frequency-powered base stations and other devices. The question is if such higher frequencies (in this review, 6–100 GHz, millimeter waves, MMW) can have a health impact. This review analyzed 94 relevant publications performing in vivo or in vitro investigations. Each study was characterized for: study type (in vivo, in vitro), biological material (species, cell type, etc.), biological endpoint, exposure (frequency, exposure duration, power density), results, and certain quality criteria. Eighty percent of the in vivo studies showed responses to exposure, while 58% of the in vitro studies demonstrated effects. The responses affected all biological endpoints studied. There was no consistent relationship between power density, exposure duration, or frequency, and exposure effects. The available studies do not provide adequate and sufficient information for a meaningful safety assessment, or for the question about non-thermal effects. There is a need for research regarding local heat developments on small surfaces, e.g., skin or the eye, and on any environmental impact. Our quality analysis shows that for future studies to be useful for safety assessment, design and implementation need to be significantly improved.

## 1. Introduction

Recent decades have experienced an unparalleled development of technologies that are categorized as information and communication technologies (ICT), which include wireless communication used for mobile telephony (MP) and e.g., Wi-Fi by using electromagnetic fields (EMF). The first generation of handheld mobile phones were available for individual, private, customers in a few countries in the late 1980’s. Subsequently, the second (2G), third (3G), and fourth (4G, LTE) generations increased their penetration rates in the society in a dramatic way, so that today there are more devices than inhabitants of the Earth. In addition, Wi-Fi and other forms of wireless data transfer have become ubiquitous, and are globally available. At present we are starting to introduce the next generation, 5G, of mobile networks. Importantly, 5G is not a new technology, but an evolution of already existing G1 to G4 technologies.

With the upcoming deployment of 5G mobile networks, significantly faster mobile broadband speeds and increasingly extensive mobile data usage will be ensured. This is made possible by the use of additional higher frequency bands. 5G is intended to be the intersection of communications, from virtual reality to autonomous vehicles to the industrial Internet and smart cities. In addition, 5G is considered the base technology for the Internet of Things (IoT), where machines communicate with machines (M2M communication). At the same time, a change in the exposure to electromagnetic fields (EMF) of humans and the environment is expected (see, for example [1,2]).

The 5G networks will work with within several different frequency bands (Table 1), of which the lower frequencies are being proposed for the first phase of the 5G networks. Several of these frequencies (principally below 1 GHz; Ultra-high frequencies, UHF) have actually been or are presently used for earlier mobile communication generations. Furthermore, much higher radio frequencies (RF) are also planned to be used at later stages of technology evolutions. The new bands are well above the UHF ranges, having wavelengths in the centimeter (3–30 GHz) or the millimeter ranges (30–300 GHz; millimeter waves, MMW). These latter bands have traditionally been used for radars and microwave links. 

The introduction of wireless communication devices that operate in the high frequency parts of the electromagnetic spectrum has attracted considerable amounts of studies that focus on health concerns. These studies encompass studies on humans (epidemiology as well as experimental studies), on animals, and on in vitro systems. Summaries and conclusions from such studies are regularly published by both national and international committees containing relevant experts (see e.g., [3,4,5]. The conclusions from these agencies and committees are that low level RF exposure does not cause symptoms (“Idiopathic Environmental Intolerance attributed to Electromagnetic Fields”, IEI-EMF), but that a “nocebo” effect (expectation of a negative outcome) can be at hand. Some studies suggest that RF exposure can cause cancer, and thus the International Agency for Research on Cancer classified RF EMF as a “possibly carcinogenic to humans” (Group 2B) [3]. In a recent recommendation of a periodically working Advisory Group for IARC “to ensure that the Monographs evaluations reflect the current state of scientific evidence relevant to carcinogenicity” the group recommended radiofrequency exposure (among others) for re-evaluation “with high priority” [6]. There is further no scientific support for that effects on other health parameters occur at exposure levels that are below exposure guideline levels, even though some research groups have published non-carcinogen related findings after RF exposure at such levels (see [4,5]). Environmental aspects of this technological development are much less investigated.

Frequencies in the MMW range are used in applications such as radar, and for some medical uses. Occupational exposure to radars have been investigated in some epidemiological studies, and the overall conclusion is that this exposure does not constitute a health hazard for the exposed personnel [7]. This is due to that exposures for all practical purposes are below the guideline levels and thus not causing tissue heating. However, further studies are considered necessary concerning the possible cancer risk in exposed workers. Medical use of MMW has been recently reviewed [8,9] suggesting a possibility for certain therapeutic applications, although the action mechanisms are unclear.

The 5G networks and the associated IoT will greatly increase the number of wireless devices compared to the present situation, necessitating a high density of infrastructure. Thus, a much higher mobile data volume per geographic area is to be created. Consequently, it is necessary to build a higher network density because the higher frequencies have shorter ranges. The question that arises, is whether using the higher frequencies can cause health effects?

Exposure limits for both the general public and occupational exposure are available and recommended by the WHO in most countries, based on recommendations from ICNIRP [10] or IEEE [11] guidelines. These limits, which have considerable safety factors included, are set so that exposure will not cause thermal damage to the biological material (thermal effects). Thus, for 10 GHz to 300 GHz, 10 W/m^2^ is recommended as the basic restriction (no thermal effects), with reference values for 400 MHz to 2 GHz (2–10 W/m^2^) and >2 GHz (10 W/m^2^). It should be pointed out that the present ICNIRP guidelines [10] are currently being revised, and new versions are to be expected in the near future. In addition, ICNIRP proposes two categories of recommendations: (1) the basic restriction values based on proven biological effects from the exposure and (2) the reference levels given for the purpose of comparison with physical value measurements. ICNIRP guidelines present no reference values above 10 GHz, only considering the basic restriction values. This is due to that only surface heating occurs since the penetration depth is so small at these frequencies. Therefore any calculations of the Specific Absorption Rate (SAR) values, that take larger volumes into consideration, are not reasonable to perform.

The SAR is the measure of the absorption of electromagnetic fields in a material and is expressed as power per mass/volume (W/kg), where the penetration depth of the electromagnetic fields depends on the wavelength of the radiation and the type of matter. The penetration depth of MMW is very shallow, hence the exposed surface area and not the volume is considered. The appropriate exposure metric for MMW is therefore the power density, power per area (W/m^2^).

It is of course too early to forecast the actual exposures to 5G networks. However, the antennas planned for 5G will have narrow antenna beams with direct alignment [12] to the receiving device. This could possibly significantly reduce environmental exposure compared to the present exposure situation. However, it is also argued that the addition of a very high number of 5G network components will increase the total EMF exposure in the environment, and that higher exposures to the higher frequencies can lead to adverse health effects.

Therefore, the question arises, what do we know so far about the effects on biological structures and on health due to exposure to the higher frequency bands (in this review we consider 6–100 GHz, since lower frequencies have been extensively investigated due to their use in already existing wireless communication networks)? Do so-called “non-thermal” effects (effects that occur below the thermal effect threshold) occur, that can lead to health effects? Is there relevant health-oriented research using the 5G technology relevant frequencies? Is there relevant research that can make a significant contribution to improving the risk assessment of exposure to the general population? Answers to these questions are necessary for a rapid and safe implementation of a technology with great potential.

## 2. Materials and Methods 

This review takes into account scientific studies that used frequencies from 6 GHz to 100 GHz as the source of exposure. The review is based on available data in the field of public literature, papers written in English until the end of 2018 (PubMed database: www.ncbi.nlm.nih.gov/pubmed), EMF-Portal (www.emf-portal.org), and other relevant literature such as documents from ICNIRP, SCENIHR, WHO, IARC, IEEE, etc.). In addition, more refined research was conducted when necessary from sources that were not included in the above-mentioned databases (relevant abstracts from conferences, abstract books, and archives of journals). The resulting studies were examined for technical and scientific data and presented in the supplementary Table S1.

As a pragmatic approach, we interpreted the results as a “response” when the authors themselves reported the result as an “effect/response” based on a statistical analysis and the *p*-value < 0.05.

Next we defined necessary criteria for study quality, both from a biomedical and physical point of view (see [13]). The results of the studies were (if possible) analysed for correlations with study quality according to the correlation approach done by Simkó et al. [14]. The studies were analysed with reference to a minimum of criteria in terms of experimental design and implementation. The following criteria were considered: were the experiments performed in the presence of an appropriate sham/exposure control, temperature control, positive control, were the samples blinded, and was a comprehensive dosimetry presented.

The study is divided into a descriptive part, which covers the description of all selected studies, their exposure conditions, frequency ranges (6 GHz to 100 GHz), dose levels, etc., as well as the biological results, presented in a Master-Table (Table S1). Review articles were not considered. The outcomes of the studies were furthermore analyzed and discussed according to frequency domains, and power density and exposure duration. If appropriate, we include an evidence-based interpretative part regarding risk from exposures according to the criteria of SCHEER [15].

## 3. Results

In the following, health-related published scientific papers dealing with frequencies from 6 GHz to 100 GHz (using the term MMW for all the frequencies) are described in detail. It should be noted that there are no epidemiological studies dealing with wireless communication for this frequency range, thus, this review will cover studies performed in vivo and in vitro. 

Thermal biological effects of radiofrequency electromagnetic fields occur when the SAR values exceed a certain limit, namely 4 W/kg (general population exposure limit: SAR 0.08 W/kg), which causes a tissue heating of 1 °C. However, in the literature, biological effects below 4 W/kg SAR values have been described. Since such effects are considered to be not due to warming, they are termed non-thermal effects. In the present review, in some individual studies, the authors interpreted thermal effects as “no effect”. Those ones and studies without response/effect of MMW exposure were considered as “no response/effect” in our present analysis.

### 3.1. Grouping of Selected Parameters

For analysis, 94 publications were identified and selected from the accessible databases (in vivo and in vitro) [16,17,18,19,20,21,22,23,24,25,26,27,28,29,30,31,32,33,34,35,36,37,38,39,40,41,42,43,44,45,46,47,48,49,50,51,52,53,54,55,56,57,58,59,60,61,62,63,64,65,66,67,68,69,70,71,72,73,74,75,76,77,78,79,80,81,82,83,84,85,86,87,88,89,90,91,92,93,94,95,96,97,98,99,100,101,102,103,104,105,106,107,108,109]. It should be noted that the total number of individual examinations is larger than the number of publications, since some authors investigated several physical and/or biological conditions in the same publication. 

Various biological endpoints have been identified, which are referred to as “response” or effects when appropriate. Since the list of these endpoints is relatively long, we have not mentioned them in detail, but summarized them in groups: Physiological, neurological, histological changes, or in in vitro studies gene or protein expression, cytotoxic effects, genotoxic changes, and also temperature-related reactions.

For a detailed analysis, a “Master-table” (Table S1) was prepared in which all parameters considered in the studies were included. The table contains the following information: frequency, in vivo or in vitro study (the latter distinguishes between primary cells and cell lines), power density, exposure duration, biological endpoints, and response. Some studies lack information on individual parameters. For example, a publication had to be excluded completely because there was no information about the frequency. In nine studies the power density data were absent and in seven studies the calculated SAR values were provided instead of the power density. In ten studies, the exposure time was not given.

The 45 in vivo studies were mainly conducted on mammals (mouse, rat, rabbit) and a few on humans. In some studies, bacteria, fungi, and other living material were also used for the experiments. 80% of all in vivo studies showed exposure-related reactions.

Primary cells (n = 24) or cell lines (n = 29) were used in the 53 in vitro studies, with approximately 70% of the primary cell studies and 40% of the cell line investigations showing exposure-related responses (Table 2).

**Figure 1:**
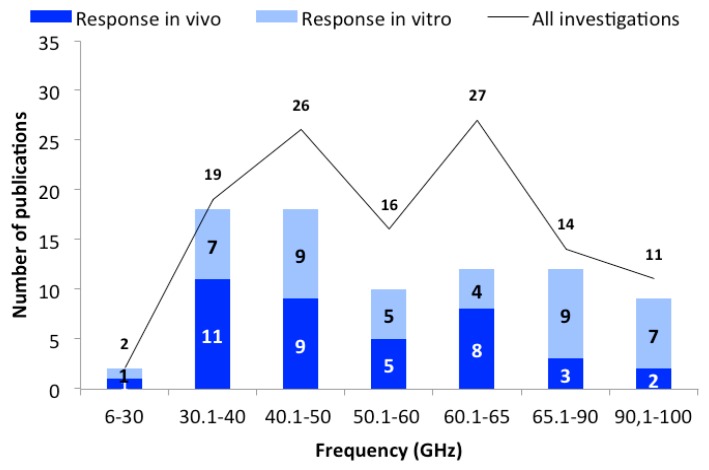
The number of publications as a function of frequency domains. The black line represents the total number of publications, and bars represent the in vivo (dark blue) and in vitro (light blue) studies with biological responses.

All identified studies were analyzed as a function of frequency. For this purpose, frequency domains (groups) have been created (Figure 1) to analyze and illustrate the results. The frequency groups from 30 to 60 GHz were grouped in ten-GHz increments (up to 30, 30.1–40, 40.1–50, 50.1–60 GHz). The frequency range 60–65 GHz was extra analyzed as in this group a larger number of publications was identified (in comparison to the other groups). Due to the low number of publications above 65.0 GHz, data was merged into the groups of “65.1–90” and “above 90 GHz”. As shown in Figure 1, the majority of studies show a frequency-independent response after MMW exposure.

#### 3.1.1. Frequency Ranges

All data regarding the individual papers are found in Table S1.

##### Up to 30 GHz

The first group “up to 30 GHz” was introduced since some of the 5G frequencies fall within this frequency range. Unfortunately, there are only two publications in this group, both showing responses to the MMW exposure. A study that was conducted on bacteria and fungi showed an increase in cell growth [58]. The other in vitro study was performed on fibroblasts (25 GHz, 0.80 mW/cm^2^, 20 min), with genotoxic effects observed at high SAR levels (20 W/kg) [24]. A graphical presentation of the outcomes is presented in Figure 1 for this and all other frequency domains.

##### Frequency Group 30.1–40 GHz

As shown in Figure 1, responses were detected in approximately 95% of the 19 studies. In all in vivo studies responses were described after exposure [25,27,36,37,55,56,78,79,87,91,103,104]. Endpoints ranged from recorded footpad edema, which is a frequent endpoint for the measurement of inflammatory responses, to morphological changes, changes in skin temperature, blood pressure, heart rate, body temperature, neuronal electrical activity, and EEG analyses. Protein expression studies, oxidative stress marker measurements, histological investigations, and induction of cell death (apoptosis) were performed. Only one study used lower power densities (0.01 mW/cm^2^, 0.1 mW/cm^2^; SAR: 0.15, 1.5 W/kg; 20 min, 40 min) to study inflammatory responses [27]. The authors determined the frequency-dependent anti-inflammatory effect as a function of power density and exposure duration and did not rule out temperature-related effects. The power densities of the other in vivo studies were extremely high (10, 75, 500–5000 mW/cm^2^), so the induced effects were likely temperature dependent.

Eight in vitro studies were performed [18,20,47,91,97,99,101,102] of which seven reported responses. In one study [99], human blood cells (*ex vivo*) were exposed to MMW for 5, 15 and 30 min (32.9–39.6 GHz, 10 mW/cm^2^). The activation of the cells was examined in the presence or absence of bacteria. It was shown that in the presence of bacterial activation and after 15 min of exposure, the cells were activated to release free radicals. These results were similar to the heated samples (positive controls), so a temperature effect is plausible. The induction of differentiation of bone marrow cells in to neuronal phenotype cells was also demonstrated (36.11 GHz, 10 mW/cm^2^, 3 × 10 min every 2 h for 24 h) [97]. In two studies, temperature-related reactions were described at the protein level [18,91]. When the cell cultures were cooled during exposure to prevent the induced temperature increase, no responses were detected.

In three publications, a research group described cell cycle changes, induction of cell death and activation of differentiation processes in primary cells (rat bone cells and mesenchymal stem cells) after exposure to 30–40 GHz (4 mW/cm^2^, different exposure durations) [47,101,102]. Unfortunately, the minimum quality criteria were not fulfilled in any of the three studies, mainly because there were no temperature controls.

##### Frequency Group 40.1–50 GHz

In the 40.1–50 GHz frequency group, 26 studies were identified, 13 in vivo [16,17,26,48,49,51,53,65,69,74,80,84,98] and 13 in vitro [29,30,31,62,64,86,89,92,93,100,105,107] with nine studies showing responses. A large number of studies have tested cell biology endpoints such as cell proliferation, gene or protein expression, and changes in oxidative stress. In addition, immunological, neurological, morphological and genotoxic effects were investigated. The power densities used vary enormously, from 0.02 to 450 mW/cm^2^, and one publication gave no information. 

In healthy volunteers, a double-blind study was performed to investigate the effects of MMW on experimentally induced cold pain (42.25 GHz, <17.2 mW/cm^2^, 30 min) [74]. The authors found no difference from the placebo effect. This study was a repeat of a previous study with volunteers and the results of the older study could not be confirmed. The other four in vivo studies with no detectable effects were investigating genotoxic effects or oxidative stress [17,48,49,98].

Five in vivo publications addressed the effects of MMW on the immune system of mice or rats, finding activation of the immune system at both the cellular and molecular levels (41.95 or 42.2 GHz, 19.5 μW/cm^2^, 0, 1, 31.5 mW/cm^2^, 20 min or intermittently over 3 days) [26,48,51,53,84].

MMW exposure of frog isolated nerve cells, (41.34 GHz, 0.02, 0.1, 0.5, 2.6 mW/cm^2^, 10–23 min) lead to a reduction of the action potential frequency. Interestingly, the effects at higher power density (2.6 mW/cm^2^) were similar to conventional heating [49].

One study detected an increase in the motility of human spermatozoa after 15 min of exposure (42.25 GHz, 0.03 mW/cm^2^) [100]. Additional in vitro tests have identified the formation of free radicals, the activation of calcium-dependent potassium ion channels (around 42 GHz, 100, 150, 240 μW/cm^2^, 20–40 min) as well as changes at the cell membrane in exposed cells [29,30,100].

No responses on cell biological endpoints (cell cycle changes, cell death, heat shock proteins) were detected in four additional in vitro studies.

##### Frequency Group 50.1–60 GHz

We identified 16 studies in the frequency group 50.1-60 GHz (six in vivo, ten in vitro) and 60% of the studies showed responses to MMW exposures [21,23,35,38,43,46,59,61,72,77,81,83,85,94,109].

In five of the in vivo studies very different responses were shown. In a study on healthy volunteers, the authors wanted to find out whether the human skin at a so-called acupuncture point has different dielectric properties during exposure to MMW. They found that these properties change during exposure to 50–61 GHz from the surrounding skin [23].

A pilot study on mice (60 GHz, 0.5 mW/cm^2^, lifelong exposure for 30 min/5 days a week) showed that MMW exposure affects cancer-induced cells and increases in motor activity of healthy mice [61].

In rats, the influence of 54 GHz, 150 mW/cm^2^, on an area of approximately 2 cm^2^ on the head was examined [81]. This transcranial electromagnetic brain stimulation induced pain prevention and prevented the conditioned avoidance response to a pain stimulus in 50% of the animals. However, no changes were detected when serotonin inhibitors were previously administered. Therefore, the authors concluded that transcranial electromagnetic brain stimulation promotes the synthesis of serotonin, a transmitter that changes the animals’ pain threshold.

The effects of MMW were also tested (60 GHz, 475 mW/cm^2^, 1.898 mW/cm^2^, 6, 30 min) on rabbit eyes, describing acute thermal injuries of various types [38]. The authors also pointed out that the higher temperature just below the eye surface could induce injury.

Neurological investigations were performed on leeches (60 GHz, 1 min, 1, 2, 4 mW/cm^2^) [77] and electrophysiological studies were performed on frog oocytes (60 GHz, up to 5 min) [85]. In both experimental systems effects were described, which were induced by the temperature rise.

Cell biological and morphological changes after exposure to 0.7–1.0 μW/cm^2^ (intermittent) were reported in three in vitro studies [72,83,94], with two publications providing no information regarding power density or exposure duration. At the level of protein analysis and total genome analysis no changes were identified in four in vitro studies [35,46,59,109].

##### Frequency Group 60.1–65 GHz

The number of studies in the 60.1–65 GHz frequency group is 27. Of these, twelve reported effects from exposure to MMW, and no responses were found in 15 studies.

The in vivo studies investigated different topics [23,27,44,52,67,68,70,71,73,75,76]. Thus, two studies examined the effects on tumor development in mice injected with tumor cells [52,70]. In one of the studies it was reported that exposure to 61.22 GHz, 13.3 mW/cm^2^, inhibited the growth of melanoma cells (exposure 15 days after tumor cell injection, 15 min/day) [70].

Other publications from one research group investigated the potential of MMW for pain relief and the associated biological mechanisms of action [67,71,73,75,76]. Several of the studies were performed on mice skin exposed to 61.22 GHz for 15 min. The most commonly used power density was 15 mW/cm^2^. Another study addressed the dose issue with no effect below 1.5 mW/cm^2^. The authors’ conclusion is that MMW can lower the hypoalgesia threshold, which is likely mediated by the release of opioids.

The effects of 61.22 GHz exposure of mice were examined also with respect to the immune system [52]. The animals were exposed on three consecutive days for 30 min per day. The exposure caused peak SAR values of 885 W/kg on the nose of the animals where the exposure took place. The power density was 31 mW/cm^2^ and the measured temperature rise reached 1 °C. It was found that MMW modulates the effects of the cancer drug cyclophosamide. In particular, the T-cell system of the immune system was activated and various other immune system relevant parameters affected.

The similar exposure condition was used in a study on gastrointestinal function, however no effects were identified [68].

A single exposure for eight hours (61 GHz, 10 mW/cm^2^), or five times four hours, did not cause eye damage to rabbits and rhesus monkeys [44]. It should be emphasized that several of the mentioned studies come from the same laboratory, and all criteria for the study quality are met. However, the authors were able to replicate their own findings on pain relief whereas other laboratories have not replicated this work. In the in vitro studies, various biological endpoints were examined [28,32,33,34,42,45,50,59,60,66,83,88,94,95,108]. 

In one study, neurons of snails (*Lymnea*) were exposed at 60.22–62.22 GHz and no non-thermal responses on the ion currents were identified [28].

In a series of investigations with nerve cell-relevant cell lines, the dopamine transmission properties, stress, pain and membrane protein expression were investigated (60.4 GHz, 10 mW/cm^2^, 24 h) and no responses were detected [32,33,34,59,60,108].

The same exposure setup has also been used in studies examining different stress response related genes (0.14–20 mW/cm^2^) [59]. No effects were found at the gene expression level. Interestingly, the overall genome impact was influenced when the exposure (60.4 GHz, 20 mW/cm^2^, 3 h) of the primary human keratinocytes was combined with 2-deoxyglucose, a glucose-6- phosphatase inhibitor. This co-exposure caused a change in the amount of six different transcription factors, the effect differing from the effect of 2-deoxyglucose alone and 60.4 GHz alone (both factors alone induced no changes).

Other studies also examined human keratinocytes and astrocytoma glial cells after exposure to 60 GHz (0.54, 1 and 5.4 mW/cm^2^) [60,108]. Various parameters such as cell survival, intracellular protein homeostasis, and stress-sensitive gene expression were investigated. Also, in these studies, no effects were observed. In contrast, in one publication, the elevation of an inflammatory marker (IL1-β) was observed in human keratinocytes after exposure (61.2 GHz, 29 mW/cm^2^, 15, 30 min), while other inflammatory markers (chemotaxis, adhesion and proliferation) have remained unchanged [95].

Another type of study was performed on rat brain cortical slices [66]. The brain slices were exposed to a field of 60.125 GHz (1 μW/cm^2^) for 1 min, and then specific electrophysiological parameters were measured. In many slices, transient responses on membrane characteristics and action potential amplitude and duration were observed. The exposure caused a temperature rise of the medium (of 3 °C) in which the sections were stored. Interestingly, a chronically induced Ca^2+^ blockade did not affect the MMW response.

##### Frequency Group 65.1–90 GHz

The studies in the frequency group of 65.1 to 90 GHz were performed both in vivo and in vitro in a total of 14 articles (four in vivo and 11 in vitro investigations). The studies vary widely, based on different hypotheses, biological endpoints, power densities, and exposure durations. In addition, some studies have used biological materials to identify physical properties such as dielectric properties and skin reflection coefficient. The latter studies are discussed in Section 4.2.

Four in vivo studies reported responses after MMW exposure. One study examined the dose of eye damage (especially damage to the corneal epithelium) [40]. The dose was calculated as DD_50_ (based on the results for which the probability of eye damage was 50%). The experiments were carried out on rats with an exposure of 75 GHz, the DD_50_ value being 143 mW/cm^2^.

Other in vivo studies were performed on rats and mice as well as on insects [27,42,57]. The study on mice used different frequencies of 37.5 to 70 GHz, with power densities of 0.01 and 0.3 mW/cm^2^ for 20 to 40 min. A single whole-body exposure of the animals reduced both the footpad edema and local hyperthermia on average by 20% at the frequencies of 42.2, 51.8, and 65 GHz. Other frequencies had no influence. 

The study on insects (*Chironomidae*) focused on DNA effects of giant chromosomes of the salivary glands of the animals with different frequencies (64.1–69.1, 67.2, 68.2 GHz) [42]. All frequencies, using power densities <6 mW/cm^2^, caused a reduction in the size of a particular area of the chromosome. This in turn led to the expression of certain secretory proteins of the salivary gland.

Different aspects were studied in the in vitro studies [18,28,39,50,64,72,83,89,94,106], where nerve cell function was investigated in three studies. Two studies used nerve cells from the snail *Lymnea* that were exposed at 75 GHz for a few minutes at very high SAR levels (up to 4200 W/kg, power density was not reported) [28,39]. The authors observed thermal effects on the ion currents and the firing rate of the action potentials. Another study also described thermal effects on transmembrane currents and ionic conductivity of the cell membrane. Again, the exposure was at very high SAR levels (2000 W/kg), and the authors emphasized the temperature dependence of the reaction.

Broadband frequencies (52–78 GHz) have been used in several publications, mainly investigating the effects on cell growth and cell morphology as well as the ultrastructure of different cell lines [50,72,83,94]. The values for the power densities were not given consistently but appear to have been very low (not higher than 1 μW/cm^2^). The results indicated the inhibition of cell growth, accompanied by changes in cell morphology.

Another group of studies used hamster fibroblasts, BHK cells, and exposed the cells at 65 to 75 GHz, with the power density reaching 450 mW/cm^2^ [18,64,89]. The authors noted the inhibition of protein synthesis and cell proliferation as well as cell death at higher power densities. In a study using human dermal fibroblasts and human glioblastoma cells, no effects at the protein level (proliferation or cytotoxicity markers) were detected (70 GHz and higher, in 1 GHz increments; 3, 70 or 94 h) [106]. Power densities varied across frequencies, ranging from 1.27 μW/cm^2^ in the lower frequency range to 0.38 μW/cm^2^ at higher frequencies.

The in vitro studies in this group are similar to the in vivo studies in their diversity. The majority of studies in which responses were reported are thermal-effects due to MMW exposure. In three studies, responses at low power densities were described, but all results were from the same laboratory, and were not replicated by others. Moreover, the quality of these studies is questionable, as the quality criteria were not met.

##### Frequency Group 90.1–100 GHz

Eight out of eleven studies in the 90.1–100 GHz frequency group are in vitro studies [22,41,57,82,90,96,106]. The three in vivo investigations addressed a variety of issues including acute effects on muscle contraction, skin-reflection properties (which are more of a dose-related than health-related issue), and skin cancer [19,54,57]. The rat skin cancer study (one to two weekly, short-term exposures at 94 GHz, 1 W/kg; DMBA-initiated animals) did not show any positive outcome [54]. Another study examined the muscle contraction of mice and described some responses [19]. Again, 94 GHz was used, but power density or SAR values were not reported.

Seven of the eight in vitro studies showed responses after MMW exposure. In some studies, primary neurons were used to study the cytoskeleton (94 GHz, 31 mW/cm^2^) [82] or specific electrophysiological parameters (90–160 GHz) [22]. In the latter study it was found that the observed responses were more likely due to interactions with the cell culture medium than with the cells, although the mechanisms of action were not clear. Other studies identified responses on the DNA integrity (100 GHz and higher) [41] or described changes in intracellular signaling pathways (94 GHz, 90–160 GHz) using different cell types [57,96]. The exposure time ranged from minutes to 24 h for partially unknown exposure values. In one study no cytotoxic influence at power density levels of a few μW/cm^2^ was detected in either normal or in tumor cells. 

#### 3.1.2. Power Densities

**Figure 2:**
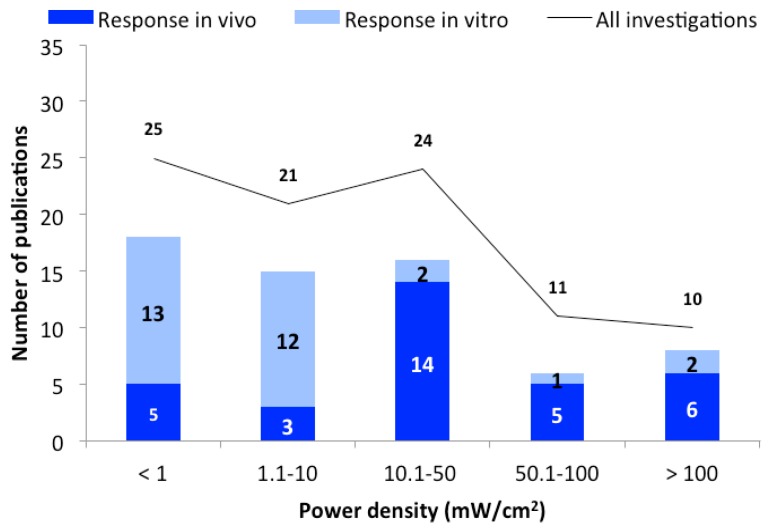
The number of publications as a function of power density. The black line represent the total number of publications, and bars represent the in vivo (dark blue) and in vitro (light blue) studies with biological responses.

All identified studies were analyzed as a function of the used power densities. The studies were grouped depending on the power density as follows: below 1; 1.1–10; 10.1 to 50; 50.1–100, and 100.1 mW/cm^2^ or higher. Studies that do not provide information on power density or SAR values are not displayed in these groups. As shown in Figure 2, the vast majority of studies show responses regardless of the power density used.

#### 3.1.3. Exposure Duration

**Figure 3:**
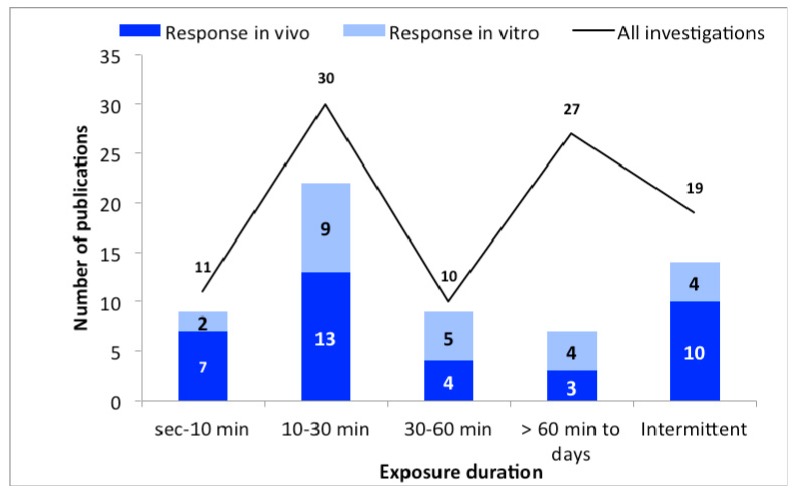
The number of publications as a function of exposure duration. The black line represent the total number of publications, and bars represent the in vivo (dark blue) and in vitro (light blue) studies with biological responses.

Exposure duration of the studies was also grouped for data analysis (Figure 3). The time groups were selected as seconds to 10 min; 10–30 min; 30–60 min; over 60 min-days and alternately/intermittently. The groups were selected so that the used exposure times and the number of studies are meaningfully summarized. Here, too, it becomes clear that the majority of all studies show responses regardless of the exposure time. Interestingly, longer exposure times (over 60 min—days) seemingly lead to fewer reactions than in the other groups.

### 3.2. Studies without Responses

Table 3 shows the number of studies in which no responses were detected after or during MMW exposure. As “no response” also such investigations were referred to, which were considered by the authors themselves as such. This means that in some cases the observed effects were described as temperature-related and not as a non-thermal MMW effect. 

Few in vivo studies have shown no response at all. Noticeable is the frequency group 40.1–50 GHz, in which 6 studies were identified. These studies investigated immunosuppression, genotoxic effects, changes in pain sensitivity, and changes in enzyme activity. One study was carried out on bacteria and fungi.

There are a variety of in vitro studies in which no responses were detected. Interestingly, studies on protein or gene expression levels often failed to detect any changes after MMW exposure. This could be due to the fact that in in vitro studies the possibility of non-thermal effects were specifically investigated, where cooling was used to counteract the temperature increase.

### 3.3. Quality Analysis 

We analyzed the quality of the selected studies according to specific criteria [14]. The studies were categorized by the presence of sham/control, dosimetry, positive control, temperature control, and whether the study was blinded. The presence of these five criteria while performing an MMW study is the minimum requirement for qualifying as a study with sufficient technical quality.

**Figure 4:**
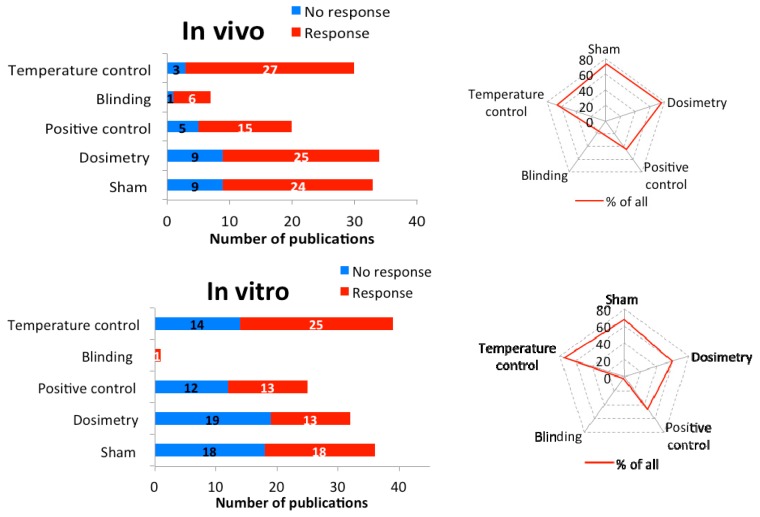
The quality of all publications: The number of in vivo (top) and in vitro (bottom) experiments (blue: no reaction, red: reaction) using the listed quality features (y-axis). The spider web shows the percentage of the quality characteristics in all examinations.

Of the 45 in vivo studies, 78% (35) demonstrated biological responses after exposure to MMW. Of all studies, 73% were performed with sham/controls, 76% employed appropriate dosimetry, 44% used positive control, and 67% were done under temperature control conditions (Figure 4). Unfortunately, only 16% of the studies were performed according to protocols that ensured blinding and only three publications were identified that met all five criteria [26,51,53]. If the blinding criterion was excluded, 13 studies could be identified that met the remaining four criteria. Considering three criteria only, namely sham, dosimetry, and temperature control, 40% (20 papers) were identified. Thus, the quality of the in vivo studies is unsatisfactory.

Out of the 53 in vitro studies, 31 showed biological responses. Only in 13 studies (42%) were three of the five quality criteria satisfied, namely the presence of sham/control, dosimetry, and temperature control (Figure 4). Positive controls were used in 47% and only one study was performed with blinded protocol (2%).

These results show that the number of examinations and the quality criteria are insufficient for a statistical analysis. It should be stressed that this quality analysis covers all publications dealing with the responses/effects of exposure to 6 to 100 GHz MMW, irrespective of the endpoints tested. To perform a correlation analysis, a larger number of comparable studies (e.g., identical endpoints in a frequency group) would be required.

## 4. Discussion

The first relevant observation during the analysis of the studies is that in most publications the aim of the investigations has been to determine the effects of MMW exposure for medical purposes. This means that the exposure devices used primarily come from medical applications (therapy or diagnostics). Very few publications dealt with health-related issues after MMW exposure in general, or with the specific topic of 5G. Therefore, the 94 publications are very heterogeneous. 

We divided the frequency bands into seven ranges and placed the studies in the relevant groups. All available information on physical and experimental parameters was collected, but the exact number of experiments in each study was not taken into account. (One publication can contain more than one experiment.) Therefore, it is the provided numbers of studies/publications that constitute the data set, not the exact numbers of experiments performed, which is significantly higher.

This report does not provide a statistical analysis of the correlation between the exposure conditions and the results, which was our original ambition. In the correlation study according to Simkó et al. [14] a frequency group was selected, with only one group of biological endpoints considered. About one hundred, exclusively in vitro, studies were identified and broken down into individual experiments in that paper. In this way, the number of experiments was sufficient to perform a correlation analysis. In the present review, the spread of biological endpoints in the individual frequency groups and the models used (in vivo and in vitro) is large and the number of studies is very low. Therefore, it was not possible to group the studies by specific endpoints and perform a statistical analysis.

Interestingly, more than half of the studies (53 publications) were conducted in the frequency bands 40.1–50 and 60.1–65 GHz (with different models and endpoints). One possible reason for this is that medical use of MMW has a long tradition in Eastern Europe. These applications use specific frequencies that fall in these two frequency groups. The studies were conducted with the aim of testing specific effects with medical relevance. In these two frequency groups, the “with responses” percentage was generally lower than in the other frequency bands (see Figure 1), where a majority of studies showed responses to exposure.

With regard to the power densities used, about half of the studies were carried out in the range up to 10 mW/cm^2^ (Figure 2). This value is ten times higher than the current ICNIRP exposure guideline [10] for the general population. Based on available data, there is no indication that higher power densities cause more frequent responses, since the percentage of responses in all groups is already at 70% (Figure 2). One exception from this high response rate is the group 50.1–100 mW/cm^2^, where the proportion of studies with reactions is slightly lower (54%). However, the total number of examinations (11) is relatively small in this group.

The results of some of the studies may suggest that exposure to power densities at or below the guideline recommendations induce biological effects. There are, however, some arguments against it. One of these is the apparent heterogeneity of the study design and the outcomes studied. There are very few (if any) independent replication studies that confirm the reported results. It is also noteworthy that there is no trend towards a classic dose-response pattern where stronger or more frequent effects would be caused by higher exposure levels. Since the studies with conditions promoting tissue warming show no greater effect than below the guideline values (1 mW/cm^2^), this would either mean that the same interactions are present at all power densities tested, or that experimental artifacts unknown to the scientists are present.

The most important physical experimental parameter is the temperature during exposure, therefore, the temperature must be consistently controlled. The need for stringent temperature control is not an insignificant or trivial matter and has been neglected or at least undervalued in many studies. Although some authors report that they performed specific temperature measurements during the experiments, this does not necessarily mean that this represents the actual temperature in the biological material. Measurements can be made, for example, in the surrounding medium but not in the exposed tissue or in the cell. It also has to be considered that the “bulk” heating (from outside to inside with a certain time course) can differ from a heating that occurs at a rather limited point (“hot spot”). In addition, the intensity of a short burst can be lost if the measurements are based on average exposure times. Such errors and problems are possible factors that have contributed to the questionable interpretation of “non-thermal effects” in some studies.

Effects after MMW exposure were shown at all exposure times with no clear time dependency. The data presented shows one exception, namely in the group “>60 min to days”, where fewer reactions were detected (Figure 3). It has to be taken into account that 27 examinations were carried out in this group, 23 of which were in vitro studies. In vitro experiments can be carried out under cooling, therefore the results can be different (see further below).

Two research groups together provide 30 of the 94 publications in the data set, and could thus possibly have a large impact on the analysis of the outcomes. One group presented at least 21 publications (42.25 and 61.82 GHz; 10 to 30 mW/cm^2^; with different exposure durations), with a variety of in vivo and in vitro studies, which mostly reported responses to exposure. The other group mainly studied gene and protein expressions (60 GHz; 5.4 to 20 mW/cm^2^; exposure durations from minutes to days) and found mainly no responses. Studies from both groups adhered well to the quality criteria in our analysis.

### 4.1. Temperature Controls in In Vitro Studies

In vivo studies that are performed within or directly on the living organism have shown both thermal and purportedly non-thermal effects after or during MMW exposure. In vitro studies are carried out on cells and most experimental parameters can be accurately set and observed. Cell cultures can thus be very carefully controlled, e.g., an induced temperature increase can be counter-cooled. Many in vitro studies considered in this review were performed using cooling of the cell culture vessels and the authors did not detect any non-thermal effects in these studies. In in vivo studies counter-cooling is not possible, thus it is very difficult to differentiate between thermal and non-thermal reactions. Therefore, in vivo and in vitro studies regarding the induced effects cannot be directly compared. An accurate dosimetry could solve this problem.

### 4.2. Dosimetry

It is important to know what the exposure of the MMW will be due to the expected introduction of a large number of 5G wireless communication devices. Given the novelty of the technology, it is currently unlikely that a large number of relevant exposure assessment studies will be available. However, an example from a recent study [110] shows that a “typical” office environment with wireless communication transmitters (5.50 GHz) leads to power densities well below the exposure guideline limits. Thus, the maximum power density was measured at 0.89 μW/cm^2^.

Partly (n = 25) the experimental studies on biological and health effects of MMW exposure are at or below the ICNIRP exposure guidelines. The power densities were often chosen so that the exposure caused no or very moderate tissue warming (<1 °C), namely in the range of 1 to 10 mW/cm^2^. Since the penetration into the tissue of these frequencies are on the order of millimeters and below, it is important to study biological effects directly or indirectly related to skin and eyes exposure. As mentioned previously, the number of available studies in the 6–100 GHz frequency range is relatively low, which is in contrast to the number of studies for lower radio frequencies. Similarly, the number of tissue dosimetry studies (especially for the skin) is very limited. However, such studies are very relevant because they show how certain exposure parameters can influence the energy input and thus the thermal behavior of the skin.

Currently, both the ICNIRP guidelines and the IEEE standards are being revised to replace the SAR values with power density above 6 GHz. However, it has already been recognized that there is a reactive near field close to the transmitter (around the antennas). Here, the energy is not radiated, but the energy envelopes the antennas. The question is whether these “reactive near fields” are important for the energy delivery to a human body near the transmitter? If this is not the case, it is sufficient to comply with the existing exposure limits based on free space power density measurements. On the other hand, a strong reactive near field would considerably complicate the exposure situation [111]. Therefore, for dosimetry modeling of distances (from the antenna) below the wavelength of the MMW (mm), temperature measurements should rather be performed in suitable phantoms rather than direct measurements of the power densities in the free space [111].

The question is how reliably the power density (in free space) can be extrapolated to possible temperature increases in human tissue? For example, Neufeld et al. [112] found that 10 GHz “bursts” (considered “safe” by ICNIRP and IEEE) can cause temperature increases of >1 °C if the burst duration is long enough. It was also discussed whether the average values of the power densities for the safety assessment are the right ones. In addition, the temperature increase by the MMW also depends on the size of the area. Thus, the factors such as the amplitude of the burst, the “averaging area” and the “averaging time” for the dosimetry would have to be considered.

Foster et al. [113] reviewed and modelled data on MMW-induced temperature increases in human skin. The model takes into account the frequencies of 3–100 GHz and smaller skin areas with the diameter of 1–2 cm. Available data on exposures lasting more than a few minutes, as well as areas of skin larger than 2 cm in diameter, were limited and made modeling difficult, but consistent with existing data. This means that this model, after appropriate evaluation for dosimetry, could use smaller areas of the skin. The authors also commented on the exposure guidelines for frequencies from 3 to 300 GHz in a separate article [114]. Based on “thermal modeling,” the authors considered the current guidelines to be conservative in terms of protection against temperature increases in the tissue. They also pointed out that the averaging time and average area provisions require further refinement and that the effects of short high intensity bursts may not be protected by the guidelines.

Zhadobov et al. [115] addressed the problem of accurate temperature measurement in in vitro MMW studies. They found that the type of thermal probe (thermocouples are better than fiber optic probes) and the size of the probe (smaller probes are more accurate) are relevant. In addition, they were able to show that the initial temperature rise during exposure is rapid (within seconds until a plateau is reached) and that the cells absorb very small amounts of energy, since most of the energy is already absorbed in the cell culture medium. Nevertheless, the authors have calculated that the exposure of 58.4 GHz with 10 mW/cm^2^ leads to SAR values of more than 100 W/kg in a cell monolayer. This value is a fraction of the SAR values of the fluid surrounding the cells.

Several studies focused on the distribution of power density and the change in skin temperature as a result of exposure to MMW in the 6 to 100 GHz frequency range. The studies are experimental and/or modeling studies using previously published data. Alekseev et al. [116,117] investigated the absorption of the skin of mice and humans at frequencies between 30 and 82 GHz (10 mW/cm^2^). They found that in both species absorption into both the epidermis and the dermis occurs with a concomitant loss of power density in the deeper regions. An extended study from the same group [118] on human forearm skin showed that both temperature increase and SAR values depend on frequency (in the interval of 25 to 75 GHz; 25, 73.3 and 128 mW/cm^2^).

Frequency dependence for temperature increases was also observed in a modeling study with human facial skin [119]. Pulsed MMWs were used (6–100 GHz, 100 mW/cm^2^, 200–10,000 ms pulse length) and the skin temperatures were modeled as the function of both pulse length and frequency. Peak skin temperature increased as a function of frequency up to 20 GHz, while above 20 GHz it proved to be dependent on “absorption hotspots”. In deeper regions (>2 mm), the temperature increases were very low and highest around 10 GHz.

In addition, certain skin constituents have been shown to affect energy absorption. It has been shown that the presence of sweat glands [120,121] and also capillaries in the dermis can cause locally elevated SAR levels [122]. The latter study showed that SAR levels in vessels could be up to 30 times higher than in the surrounding skin, depending on the diameter of the vessels.

Both [23] and [123] have reported that the dielectric properties of different areas of the skin differ. The first study found that so-called acupuncture points in healthy volunteers show different dielectric properties when exposed to MMW (50–75 GHz, 14 mW/cm^2^), while the second study even found differences between the epidermis and dermis (0–110 GHz).

These studies suggest that both the frequency and the specific condition and composition of the skin are relevant for tissue dosimetry. However, too few and very different studies are available to give a conclusive picture on dosimetry of 5G-relevant MMW exposures.

### 4.3. ICNIRP and other Exposure Recommendations

The guidelines for exposure limits for radiofrequency electromagnetic fields from 3 to 300 GHz in many countries are based on the recommendations of the International Commission on Non-Ionizing Radiation Protection (ICNIRP) [10]. However, there are also other organizations dealing with limit values such as the Institute of Electrical and Electronics Engineers, IEEE [11] or the US Federal Communications Commission, FCC [124].

The guidelines contain basic exposure limits that are indicated as SAR or power density. The limits for a given frequency differ only slightly, if at all, between the different guidelines. However, an important difference between the guidelines concerns frequency, as the SAR basic restriction values change to power density. This frequency (range) is currently set by ICNIRP at 10 GHz, while IEEE and FCC see this between 3–6 GHz. The current revision of these guidelines aims to harmonize these frequencies.

The exposure limits specified in the guidelines should protect against warming of tissue above 1 °C. The reason is that the perceived dangers of MMW energy are associated with excessive heating, called thermal effects. However, it must be considered that the guidelines mean a temperature increase of 1 °C relative to the starting temperature, regardless of the starting temperature. Elevations in temperature may cause pain in the skin when moderately increased, whereas at temperatures of 43–44 °C it may even induce burns [124,125].

At present, only thermal effects due to high-frequency electromagnetic fields are recognized as effects. This means that effects have a thermal component even if it is obviously not due to tissue that has been damaged by excessive heating. On the other hand, it has been suggested that the MMW exposure may also cause non-thermal effects. So far, however, no recognized expert committee has supported such an assertion.

### 4.4. Knowledge Gaps and Research Recommendations

Exposure of humans can occur through 5G devices with frequencies above 6 GHz, and may be primarily on the skin and, to a lesser extent, on the eyes. This is due to the very low penetration depth of this MMW. Therefore, it is important to investigate whether there are any health-related effects on the skin and/or effects associated with the skin. These include acute skin damage from tissue heating (burns), but possibly also less acute effects (such as inflammation, tumor development, etc.). Such effects could appear after prolonged and repeated heating of superficial structures (the skin). This would mean that thermal effects occur that are not due to acute but to chronic damage.

It may also be that local exposure causes energy deposition in the dermis of the skin, which may be so great as to affect nerve endings and peripheral blood vessels through warming mechanisms. Such scenarios were proposed by Ziskin [9] based on a series of studies by his group. These studies typically used exposures around 60 GHz at a power density of 10 mW/cm^2^ on the skin in the sternum area to produce systemic effects. The aim was to treat certain diseases and complaints. The idea was that the treatment induces the release of the body’s own opioids and additionally stimulates the peripheral nerves. The stimulation would depend on a local thermal effect, which, due to the frequencies, induces locally high SAR values, even at low power densities, thus warming the tissue.

Due to the contradictory information from various lines of evidence that cannot be scientifically explained, and given the large gaps in knowledge regarding the health impact of MMW in the 6–100 GHz frequency range at relevant power densities for 5G, research is needed at many levels. It is important to define exact frequency ranges and power densities for possible research projects. There is an urgent need for research in the areas of dosimetry, in vivo dose-response studies and the question of non-thermal effects. It is therefore recommended that the following knowledge gaps should be closed by appropriate research (the list of research recommendations is not prioritized):

- Exact dosimetry with consideration of the skin for relevant frequency ranges, including the consideration of short intense pulses (bursts)
- Studies on inflammatory reactions starting from the skin and the associated tissues
- In vivo studies on the influence of a possible tissue temperature increase (e.g., nude mouse or hairless mouse model)
- In vivo dose-response studies of heat development
- Use of in vitro models (3D models) of the skin for molecular and cellular endpoints
- Clarification of the question about non-thermal effects (in vitro)

There are also questions about the environmental impact, with potential consequences for human health. Since many MMW devices will be installed in the environment, the impact of MMW on insects, plants, bacteria, and fungi is relevant to investigate. Particularly relevant is the question of temperature increase in very small organisms, as the depth of penetration of the MMW could warm the whole organism.

An unrealistic scenario, however, is that MMW exposures at realistic power densities could cause systemic body warming in humans. Any local heat exposure would be dissipated by the body’s normal heat regulation system. This is mainly due to convection caused by blood flow adjacent to the superficial skin areas where the actual exposure takes place.

In summary, it should be noted that there are knowledge gaps with respect to local heat developments on small living surfaces, e.g., on the skin or on the eye, which can lead to specific health effects. In addition, the question of any possibility of non-thermal effects needs to be answered. 

## 5. Conclusions

Since the ranges up to 30 GHz and over 90 GHz are sparingly represented, this review mainly covers studies done in the frequency range from 30.1 to 65 GHz.

In summary, the majority of studies with MMW exposures show biological responses. From this observation, however, no in-depth conclusions can be drawn regarding the biological and health effects of MMW exposures in the 6–100 GHz frequency range. The studies are very different and the total number of studies is surprisingly low. The reactions occur both in vivo and in vitro and affect all biological endpoints studied.

There does not seem to be a consistent relationship between intensity (power density), exposure time, or frequency, and the effects of exposure. On the contrary, and strikingly, higher power densities do not cause more frequent responses, since the percentage of responses in most frequency groups is already at 70%. Some authors refer to their study results as having “non-thermal” causes, but few have applied appropriate temperature controls. The question therefore remains whether warming is the main cause of any observed MMW effects?

In order to evaluate and summarize the 6–100 GHz data in this review, we draw the following conclusions:

- Regarding the health effects of MMW in the 6–100 GHz frequency range at power densities not exceeding the exposure guidelines the studies provide no clear evidence, due to contradictory information from the in vivo and in vitro investigations.
- Regarding the possibility of “non-thermal” effects, the available studies provide no clear explanation of any mode of action of observed effects.
- Regarding the quality of the presented studies, too few studies fulfill the minimal quality criteria to allow any further conclusions.

## Figures and Tables

**Table 1:** Subdivision of the 5G frequency spectrum.

Frequency Range	Use	Comments
<1 GHz	Net coverage, IoT	Already partly used for earlier MP generations, longer range coverage, less costly infrastructure
1–6 GHz	Net coverage, IoT, capacity for data transfer	More spectrum available, shorter range and reduced performance compared to higher frequencies
>6 GHz	Capacity for very high data transfer	Short range, allows high speed data transfer and short latency times

**Table 2:** Overview of the total number of publications examinations.

All Publications (94)	No Response	Response	All
In vivo	10	35	45
In vitro	22	31	53
Primary cells	6	18	
Cell lines	16	13	

**Table 3:** Studies without responses.

Frequency (GHz)	No Response	
	In Vivo	In Vitro
Up to 30	0	0
0.1–40	0	2
40.1–50	6	4
50.1–60	1	5
60.1–65	2	10
65.1–90	0	6
90.1–100	1	1

## Supplementary Materials

The following are available online at https://www.mdpi.com/1660-4601/16/18/3406/s1, Table S1: Master-table of the selected (in vivo and in vitro) studies and the extracted physical, biological, and quality parameters.
