# Supplementary material for: 5G Wireless Communication and Health Effects—A Pragmatic Review Based on Available Studies Regarding 6 to 100 GHz
Source: Int J Environ Res Public Health. 2019 Sep 13;16(18):3406. doi: 10.3390/ijerph16183406 (PMC6765906; doi:10.3390/ijerph16183406)
Supplement: Supplementary file 1 [file ijerph-16-03406-s001.pdf]

5G Wireless Communication and Health Effects—A Pragmatic Review Based on Available Studies Regarding 6 to 100 GHz

TableS1. Master-table of the selected (*in vivo* and *in vitro*) studies and the extracted physical, biological, and quality parameters.

Reference	Animal/tissue	Primary cell	Cell line	Frequency	Power density	SAR (W/kg)	Exposure duration	Endpoint	Effects	Sham	Dosimetry	Positive control	Blind	Temperature control
[1]	Murine sural nerve			42.25 GHz	10–30 mW/cm^2, 45 mW/cm^2		20 s to 10 min	Electrical activity	Yes	No	Yes	No	No	Yes
[2]	Forearm and middle finger skin			42.25 GHz	208 and 55 mW/cm^2		Up to 10, 20 min	Blood flow measurements	No	No	Yes	No	No	Yes
[3]		Lymnaea neurons		60.22–62.22 GHz and 75 GHz		0–2400 W/kg	5 min, 10 min and 20 min	Ionic current	No	Yes	Yes	No	No	No
[4]		BP-4 pacemaker neuron of the pond snail *Lymnaea stagnalis*		75 GHz		600 to 4200 W/kg	12-22 min	Firing rate	No	Yes	Yes	No	No	Yes

[5]	Bilayer lipid membranes		53-78 GHz; cw or square wave pulsed	Output power peak 20 mW	2000 W/kg		Transmembrane currents and ion channel conductance	Yes	No	Yes	No	No	Yes
[6]	DBA2 and Swiss mice		60 GHz	0.5 mW/cm^2		30 min/session, 5 consecutive days/week until death	Cancer	Yes	No	Yes	No	No	Yes
[7]		RPMI 7932 human melanoma cell line	53.57-78.33 GHz wide-band frequency range, 51.05 GHz and 65.00 GHz	Not given		1 h, every second day, 3 h every day, up to 7 days	Proliferation, morphology	Yes	No	No	No	No	No
[8]		MCF-7 Human Breast Cancer Cells	52-78 GHz	0.07 μW/cm2		Not given	Morphology/TEM	Yes	No	No	No	No	No
[9]		K562	53–78 G Hz	1 μW/cm^2		1 h every other day for 8 days	Proliferation behavior, the ultrastructural morphology and the glycolitic metabolism	Yes	No	No	No	No	No
[10]		RPMI7932	42.20 GHz; 53.37 GHz	1 mW/cm^2		1 h per day; 3 or 4 days	Cell proliferation - doubling time; cell cycle progression	No	Yes	Yes	No	No	Yes

[11]		BHK-21/C13 cells	38-48 GHz; 65-75 GHz, both at 0.1 GHz increments	0-354 mW/cm^2, 0-584 mW/cm^2		1 h	Protein synthesis	No	Yes	Yes	No	No	Yes
[12]	Mouse toe, electrically stimulated		94 GHz	Not given		2, 25 min	**Skeletal muscle contraction**	Yes	Yes	No	Yes	No	Yes
[13]		HaCat Human Keratinocytes	30.16 GHz	1.0 and 3.5 mW/cm^2		1 h	Gap junction	Yes	Yes	Yes	Yes	No	Yes
[14]	Artificial axon consisting of a series of RC networks. Each network contains lipid vesicles filled with K+, causing a gradient of K+ across the lipid membrane.		53.37 GHz		1.1 W/kg	Real-time measurements for 30 min	Generation and appearance of action potentials; K+ efflux from vesicles, potentiation of valinomycin (K+ carrier) activity	Yes	Yes	Yes	No	No	Yes
[15]	Ganglion neurons of Helix pomatia and adult Wistar rats		90–160 GHz		1.49 W/kg	1, 5, and 10 min	Medium as the target of effects	Yes	Yes	Yes	Yes	No	Yes

[16]	Human forearm skin in 10 healthy volunteers			50-75 GHz was scanned in 1 GHz increments	14 mW/cm²			Dielectric properties at different frequencies in forearm skin corresponding to one acupuncture point and two control sites	Yes	No	No	No	Yes (single blind)	No
[17]			Fetal HFFF2, adult HDF	25 GHz	0.80 mW/cm²	20 W/kg	20 min, at RT	DNA breaks (comet assay, g-H2AX phosphorylation); chromosome damage (MN-CREST); malsegregation - non disjunction (ND-FISH); telomere length modulation; apoptosis induction; cell cycle stages; prosurvival gene signalling, morphology and ultrastructure	Yes	Yes	Yes	No	No	Yes
[18]	Sprague-Dawley rat			35 GHz	75 mW/cm²	13 W/kg	Until death (32-69min, 49 min)	Cardiovascular changes	No	Yes	Yes	No	No	Yes
[19]	Normal BALB/c mice and mice with peritoneal inflammation			42.2 GHz	0.1 mW/cm²	1.5 W/kg	20 min	Fatty acids in inflammation	Yes	Yes	Yes	Yes	Yes	Yes

[20]	NMRI mice		37.5 – 70 GHz, 42.2 GHz	0.01 mW/cm^2, 0.1 mW/cm^2	0.15, 1.5 W/kg	20 min, 40 min	Footpad edema	Yes	Yes	Yes	Yes	No	Yes
[21]		Neutrophils of NMRI mice	41.75–42.15 GHz, 41.95 GHz	150 μW/cm^2, 240 μW /cm^2,		40 min	ROS	Yes	No	Yes	No	No	No
[22]		Vero (green african marmoset) kidney cells	42.25 GHz	100 μW/cm^2		20-30 min	Activity of calcium-depending potassium-ion channels	Yes	Yes	No	No	No	Yes
[23]	The yeast *S. Cerevisiae*		48.6-48.9 GHz	2 mW/cm^2		Over night	Cell growth (absorbance at 525 nm)	Yes	No	No	No	No	Yes
[24]		PC12	60.4 GHz	10 mW/cm^2		24 h	Protein expressions of HSP70, TRPV1, TRPV2, and P2 3	No	Yes	Yes	Yes	No	Yes
[25]		Rat adrenal medulla PC12 cells treated with NGF	60.4 GHz	10 mW/cm^2		24 h	Dopamine levels and turnover (DOPAC levels and dopamine/DOPAC ratios); DAT (dopamine transporter) expression levels	No	Yes	Yes	Yes	No	Yes
[26]		Rat adrenal medulla PC12 cells, subclone Neuroscreen -1	60.4 GHz	10 mW/cm^2		24 h	Membrane and stress related protein expression	No	Yes	Yes	Yes	No	Yes

[27]		Human keratinocytes		60 GHz	20 mW/cm²	594 W/kg	1-3 h, 3 h	Whole gene expression approach	No	Yes	Yes	Yes	No	Yes
[28]	Albino mice and C57Bl/6 mice			37 GHz	10 mW/cm²		3.5 h/day for one month	Pathomorphological changes	Yes	No	No	No	No	No
[29]	Sprague-Dawley rats			35-GHz	75 mW/cm²		19.3±1.3, 38.2±2.7 min	Pathophysiology, whole body heating effects	No	Yes	Yes	No	No	Yes
[30]	Dutch rabbit, eye			60 GHz	475 mW/cm², 1.898 mW/cm²		6, 30 min	Acute ocular injury	No	Yes	Yes	No	No	No
[31]	Male Dutch-belted pigmented rabbits			40 GHz; 75 GHz; 95 GHz.	10-400 mW/cm²		6 min; 30 min	Corneal epithelial damage	Yes	Yes	No	Yes	No	Yes
[32]		Human lymphocytes		100 GHz	0.031 mW/cm²	2.4 W/kg	1, 2 and 24 h a	Genetic effects, proliferaton	Yes	Yes	Yes	No	No	No
[33]	*Acricotopus lucidus* (Chironomidae) salivary gland giant chromosomes			64.1-69.1 GHz (sweep, sweep time 6 s); 67.200 GHz; 68.200 GHz	≤6 mW/cm²		2 h	Chromosome puff sizes	Yes	Yes	Yes	No	Yes	Yes
[34]			Human corneal epithelial (HCE-T) and human lens epithelial (SRA01/04) cells	60 GHz	1 mW/cm²		24 h	Micronucleus formation, DNA strand breaks, HSP expression	No	Yes	Yes	Yes	No	Yes

[35]	Rabbit, Rhesus monkey (Macaca mulatta), Eye			61 GHz	1 mW/cm^2		8 h, 5 x 4 h on consecutive days	Ocular injury	No	Yes	Yes	No	No	No
[36]		Primary human skin		60.4 GHz	1.8 mW/cm2	42.4 W/kg	1, 6, or 24 h	Whole-Genome Expression Analysis	Yes	Yes	Yes	No	No	Yes
[37]			Human melanoma cell line A375 and the human keratinocyte cell line, HaCat	60 GHz	1, 20 mW/cm^2		20 min, 1 h, 6 h,16 h, or 24 h	Endoplasmic reticulum stress sensor gene expression	No	Yes	Yes	Yes	No	Yes
[38]		SD rats chondrocytes		30-40 GHz	4 mW/cm^2		4 x for 15, 30 or 60 min every 12 h	G1/S cell cycle progression	Yes	Yes	No	No	No	No
[39]	SKH-1 mouse, midback, blood			42.2 GHz	31 mW/cm^2	622 W/kg	30 min/d, 5 d/week for 3 weeks	Catalase, also pretreated with cyclophosphamide (CPA)	No	Yes	Yes	Yes	No	No
[40]	Mouse/BALB/c, nose area			42.2 GHz	32 mW/cm^2	622 W/kg	30 min/day for 3 days	CPA induced leukopenia and myelosuppression	No	Yes	Yes	Yes	No	No
[41]	BALB/c mouse, nose			42.2 GHz	31 5 mW/cm^2	622 100 W/kg	30 min daily for 3 days	T cell activation, proliferation, and effector functions in pretreated with cyclophosphamide	Yes	Yes	Yes	Yes	Yes	Yes

						(CPA)						
[42]	BALB/c mouse, nose	61.22 GHz	31 5 mW/cm^2	885 100 W/kg	30 min/day for 3 consecutive days	Modulation of the effect of cyclophosphamide (CPA)	Yes	Yes	Yes	No	No	Yes
[43]	BALB/c mouse, nose	42.2 GHz	31 5 mW/cm^2	623 100 W/kg	31 min daily for 3 days	Pretreated with CPA, cell viability/cell division/proliferation: cytotoxicity, effects on the immune system: natural killer cell activation (flow cytometry; expression of CD69); cytotoxicity); cytokine measurement (TNF-alpha and IFN-gamma)	Yes	Yes	Yes	Yes	Yes	Yes
[44]	(DMBA)-induced SENCAR mouse	94 GHz	1000 mW/cm^2, 333 mW/cm^2		10 sec, 10 s/week for 12 weeks	Skin carcinogenesis	No	Yes	Yes	Yes	No	Yes
[45]	Sprague-Dawley rats	35 GHz, 94 GHz	75 mW/cm^2, 90 mW/cm^2		60-70 min	Temperature changes in skin, subcutis, and colon, and the time to reach circulatory collapse (mean arterial blood pressure, 20 mm Hg)	Yes	Yes	Yes	Yes	No	Yes

[46]				35 GHz	75 mW/cm²		Until the colonic temperature reached 41– 42 C (ca. 60 min)	Gene expression, histophathology	No	Yes	Yes	Yes	No	Yes
[47]	160 adult (3 months old) male albino rats.	Heart muscle cells/tissue		90–160 GHz		1.49 mw/g	10, 15 min	[3h]-ouabain binding with cell membrane, 45ca2+ uptake and intracellular cyclic nucleotides contents	Yes	Yes	No	Yes	No	Yes
[48]	Branhamella catarrhalis ATCC 23246, Kocuria rosea CIP 71.15T, Staphylococcus aureus CIP 65.8, ATCC 25923, Streptomyces griseus ATCC 23915 bacterial strains, and the yeast Saccharo-myces cerevisiae			18 GHz		3 kw/kg and 5 kw/kg	Up to 1 h	(46 nm) nanopsheres uptake	Yes	Yes	Yes	Yes	No	Yes

[49]		Human glial U-251 MG,	59.16, 60.43, and 61.15 GHz, 59.87 and 60.83 GHz	0.14 mW/cm²	2.64 – 0.08 to 3.3 – 0.1 W/kg	24 h	Expression of endogenous ER-stress biomarkers, namely, the chaperones bip/GRP78 and ORP150/GRP170, qpcr	No	Yes	Yes	Yes	No	Yes
[50]		Human glial U-251 MG,	60.4 GHz	0.14 mW/cm²		24, 48, or 72 h	ER protein folding and secretion, induction of XBP1 or ATF6 transcription factors maturation, expression of ER-stress sensor, bip/GRP78	No	Yes	Yes	Yes	No	Yes
[51]	Frog isolated nerve (n. Ischiadicus + n. Peroneus)		41.34 GHz + high-rate electrical stimulation of the nerve (I-IRS, 20 twin pulsess-‘,9msinter-pulseinterval)	0.02, 0.1, 0.5, or 2.6 mW/cm²		23 min	Action potential	Yes	Yes	Yes	No	No	Yes
[52]	Isolated frog sciatic nerve		40–52 GHz	0.24–3.0 mW/cm², 2–3 mW/cm²		10–60 min	Action potential	Yes	Yes	Yes	No	No	Yes
[53]		BHK-21/C13 cells	41.8 GHz; 74.0 GHz	320 mW/cm², 450 mW/cm²		1 h	RNA and protein synthesis; cell morphology	Yes/ no.	Yes	Yes	No	No	Yes

[54]	20 healthy male volunteers		42.25 GHz (active generator); mw within 50–75 GHz frequency range (noise generator)	<17.2 mW/cm²	30 min	Pain threshold, measured using the cold pressor test cpt and heart rate, blood pressure, incidence of subjective sensations (paresthesia) during exposure, as well as quality of volunteers' blinding	No	Yes	No	No	Yes	No
[55]	Organotypic cortical slices	Organotypic cortical slices	60.125 GHz	1 μW/cm2	1 min	Action potential	Yes	No	No	Yes	No	Yes
[56]	Swiss Webster outbred mice		61.22 GHz	15 mW/cm²	15 min	Hypoaglesia	Yes	Yes	Yes	Yes	No	Yes
[57]	Swiss Webster outbred mice		61.22 GHz	15 mW/cm²	15 min	Gut transit and colorectal passage	No	Yes	Yes	Yes	No	Yes
[58]	Swiss Webster mice, nose		42.25, 53.57, and 61.22 GHz	13.3 mW/cm²	15 min	Hypoaglesia	Yes	Yes	Yes	Yes	No	Yes
[59]	Swiss Webster outbred mice, head/nose		61.22 GHz	13.3 mW/cm²	15 min, 15 min/day for 5 days	Melanoma	Yes	Yes	Yes	Yes	No	Yes
[60]	Swiss albino mouse, plantar surface of hind paw		61.22 GHz	15 mW/cm²	15 min	*Hypoalgesia, Cold-water tail flick test*	Yes	Yes	Yes	No	No	Yes

[61]	Swiss albino mouse, nose; glabrous skin of right paw; hairy skin of the mid back			61.22 GHz	15 mW/cm²		15 min	*Hypoalgesia, Cold-water tail flick test*	Yes	Yes	Yes	No	No	Yes
[62]	Humans			42.25 GHz	25 mW/cm²		30 min	Suppression of pain sensation	Yes	Yes	Yes	Yes	Yes	No
[63]	Male Swiss albino mice			61.22 GHz	15 mW/cm²		15 min	Scratching frequency during 90 min after s.c. Injection of pruritus (itching) agent 48/80.	Yes	No	Yes	No	No	Yes
[64]	Male Swiss albino mice			61.22 GHz	0.15, 0.5. 1.5, 5.0 mW/cm²		15 min	Tail withdrawal as pain response in cold water flick test	Yes	Yes	Yes	No	No	Yes
[65]	Leech	Individual neurons in the leech midbody ganglia		60-GHz	1, 2, and 4 mW/cm²		1 min	Real-time effects on action potential, and as "positive control" bath heating	Yes	No	No	Yes	No	Yes
[66]	Male Sprage-Dawley rats			35 GHz	75 mW/cm²	13 W/kg	Until mean arterial pressure dropped to 75 mm Hg (36.7 +/- 0.7 min)	Mean arterial pressure, heart rate, body temperature	Yes	No	Yes	No	No	Yes
[67]	Male Sprage-Dawley rats			35 GHz	75 mW/cm²	13 W/kg	Until mean arterial pressure	Mean arterial pressure, heart rate, body temperature	Yes	No	Yes	No	No	Yes

					dropped to 75 mm Hg (36.7 +/- 0.7 min)							
[68]	NMRI mice, peritoneal neutrophils	41.95 GHz	19.5 μW/cm2	0.45 W/kg	20 min at 20 oc	ROS production	Yes	No	Yes	No	No	No
[69]	Male Wistar rats	54 GHz	150 mW/cm²		10 min	Conditioned avoidance response to induced pain stimulus	Yes	No	No	No	No	No
[70]	Xenopus laevis embryonal spinal cord neurons	94 GHz	31 mW/cm²		Seconds	Microtubule assembly/disassembly	Yes	Yes	No	Yes	No	Yes
[71]	Male Wistar rats	42.2 GHz	Not provided	Not provided	2 × 4o min with 40 min interval before and after electric stimulation	C-Fos activation in hypothalamus; NK cell activtiy in spleen	Yes	No	No	No	No	No
[72]	Xenopus laevis oocytes	60 GHz	0.18–6 mw/mm2 at the bottom of the oocyte and 0.01–0.33 mw/mm2 at the top		2h	Membrane excitability protein expression by electrophysiological recordings	Yes	No	Yes	Yes	No	Yes

[73]		Human buccal epithelium cells from female donors		42.2 GHz	0.2 mW/cm²	5 sec	Electronegativity of cell nuclei; presence of heterochromatin granules in nuclei	Yes	No	Yes	No	No	Yes
[74]	Male Wistar rats			40 GHz	Not given	3 x 10 min, 10 min interval	Electric activtiy in spinal trigeminal nnucleus neurons	Yes	No	No	No	No	No
[75]		Primary human neonatal keratinocytes	HACAT	60.4 GHz	20 mW/cm²; co-exposure with a glycolysis inhibitor, 2-deoxyglucose (2dg, 20 mm)	3 h	Whole genome and ATP content	Co-exp. Yes	Yes	No	No	No	Yes
[76]			BHK-21/C13 cells	41.8 or 74.0 GHz	320 or 450 mW/cm², with or without cooling (42°C); 600, 800, and 1,000 mW/cm²	1 h	Morphological changes investigated by Scanning and Transmission Electron Microscopy	Yes/ no	Yes	Np	No	No	Yes
[77]			Mouse embryonal carcinomacell line P19 induced to neural differentiation, human epidermal keratinocytes HEKN,	94 GHz	Not given	30 min, 60 min	Intracellular Ca2+ oscillations, ATP release, NO production	Yes	Yes	No	No	No	Yes

			alone and as as co-culture											
[78]	Plasma from exposed Sprague-Dawley rats		Rat NR8383 macrophage cells	35 GHz	75 mW/cm^2		46 min (until core temperature reached 41 °C in exposed rats)	Protein expression changes and oxidative stress marker 3-nitrotyrosine (3-NT)	Yes	Yes	Yes	Yes	No	Yes
[79]		Human peripheral blood (froozen)	Human epidermal keratinocytes - hacat; human T-lyphoblast Jurkat cells: mouse B16F10 melanoma cells	42.25 GHz	34.5 mW/cm^2, 0.55 W/cm^2, 1.23 W/cm^2		30 min	Coagulation (human whole blood, blood cell components, plasma, HaCat cells); apoptosis marker expression (Annexin V staining, phosphatidyl serine externalization)	Yes	Yes	No	Yes	No	Yes
[80]			Human epidermal keratinocytes - HaCat	42.25 GHz (high power MMW exposure), 61.2 GHz (low-power MMW exposure)	29 mW/cm^2, 1670 mW/cm^2	770 W/kg, 37 kw/kg	30 min (chemokine production, gap-junction communication), 60 min (viability, Hsp70	Chemokine production (RANTES, IP-10), viability, Hsp 70 production, gap-junction communication	No	Yes	Yes	Yes	No	Yes

							production)							
[81]			Human epidermal keratinocytes - HaCat	61.2 GHz	29 mW/cm^2	770 W/kg	15 or 30 min, at room temperature	Proliferation, adhesion, chemotaxis (spontaneous or cytokine-induced), IL-1beta production	Yes	Yes	Yes	Yes	Yes	Yes
[82]			Mouse P19 embryonal carcinomacells induced to neuronal differentiation with retinoic cells. Human fibrosarcoma HAT-1080 cells.	94 GHz	310, 78, and 186 mW/cm^2		60 min P19 cells. 30 min H-1080 cells.	P19 cells - Ca2+ oscillations in real time in the presence of absence of inhibitors of N-type Ca2+ channels, Ca2+-atpase, phospholipase C. NO production. H-1080 cells actin structure and cellular mechanics.	Yes	No.	Yes.	Yes	No	Yes
[83]	Rat	Bone marrow stem cells (BMSC)		36.11 GHz	10 mW/cm^2		3 × 10 min every 2 h following beta-mercaptoethanol treatment for 24 h	Differentiation into nerve cells from BMSC	Yes	No	No	No	No	No

[84]	BALB/c mouse, nose			42.2 GHz	31,5 mW/cm²	622 W/kg	30 min/day for 3 consecutive days	Genotoxicity, also with preatreated with cyclophosphamide (CPA)	No	Yes	Yes	Yes	No	No
[85]	Human whole blood or isolated neutrophils in plasma			32.9-39.6 GHz	10 mW/cm²	145 w/kg	5, 15, 30 min	Ros generating activity seen as luminol-dependent chemoluminescence; myeloperoxidase activity; neutrophil morphology typical for activated cells	Yes	No	No	Yes	No	Yes
[86]		Cryoperserved spermatozoa		42.25 GHz	0.03 mW/cm²		5, 10, 15 min	Motility, membrane integrity, chromatin decondensation, apoptosis (Annexin V)	Yes	No	No	No	No	No
[87]		Chondrocytes		30-40 GHz	4 mW/cm²		30 min/day for three consequitive days; 60 min/day for three consequitive days	Apoptosis (Annexin V, mitochondrial membrane potential, cytochrome c, caspase-3 activation)	Yes	No	No	Yes	No	No

[88]	Sprague-Dawley rats	Mesenchymal stem cells		30-40 GHz	4 mW/cm^2		30 min/day for 1, 4, 8 or 12 days; 60 min/day for 1, 4, 8 or 12 days	Chondrocyte differentiation (Cbfa1 and Sox9 mra expression, cellular morphology, heterochromia, collagen II expression	Yes	No	No	No	No	No
[89]	New Zealand White rabbits (male and female)			37.5 GHz	10 mW/cm^2		20 or 40 min per day for 10 days, six weeks after surgery	Histologic appearance (Modified Mankin Score (MMS); chondrocyte apoptosis; caspase-3, caspase-8, and MMP-13 expression	Yes	Yes	No	No	No	No
[90]	Male Sprage-Dawley rats			35 GHz	500, 900, 1200, 1500 mW/cm^2 at 3.6 cm2 (irradiated area), 1500, 3500, 5000, 7500 mW/cm^2 at 1.8 cm^2		30 s	EEG wavelet analysis documenting stress reactions	Yes	Yes	No	No	No	Yes
[91]			Normal human skin fibroblast (NB1RBG) and human glioblastoma (A172) cells	Sweeping from 70 GHz to 300 GHz in 1.0 GHz steps.	10 μW, 1.27 μW/cm^2 at 100 GHz and 0.38 μW/cm^2 at 300 GHz.		3, 70 or 94 h	Proliferation, and MTS [3-(4,5-dimethylthiazol-2-yl)-5-(3-car-boxymethoxyphenyl)-2-(4-sulfophenyl)-2H-tetrazolium, inner salt] was used for cell activity and cytotoxicity assays	No	Yes	No	Yes	No	Yes

[92]	E. Coli xl-1 blue		42 GHz	2.6 mW/m², 32 mW/m²		40 min	Growth rate, absorption spectra at 41-42 GHz, dna plasmid transformation efficiency	No	No	Yes	No	No	No
[93]		Keratinocytes HaCat, astrocytoma glial cell line U-251 MG	60.42 GHz	1 mW/cm²	Between 17 and 21.4 W/kg for 6-well and between 13.7 and 26.2 W/kg for 96-well tissue culture plates		Cell viability and intracellular protein homeostasis	No	Yes	Yes	Yes	No	No, but calculated
[94]		U-251 MG glial cell	60 GHz	5.4 mW/cm² or 0.54 mW/cm²		1 – 33 h	Gene expression of molecular chaperones clusterin and Hsp70; specifically transcription activation, mRNA levels, protein levels	No	Yes	No	Yes	No	No

[1] S. I. Alekseev, O. V Gordiienko, A. A. Radzievsky, and M. C. Ziskin, “Millimeter wave effects on electrical responses of the sural nerve in vivo.,” *Bioelectromagnetics*, vol. 31, no. 3, pp. 180–90, 2010.

[2] S. I. Alekseev, A. A. Radzievsky, I. Szabo, and M. C. Ziskin, "Local heating of human skin by millimeter waves: Effect of blood flow," *Bioelectromagnetics*, vol. 26, no. 6, pp. 489–501, Sep. 2005.
[3] S. I. Alekseev and M. C. Ziskin, "Effects of millimeter waves on ionic currents of Lymnaea neurons," *Bioelectromagnetics*, vol. 20, no. 1, pp. 24–33, 1999.
[4] S. I. Alekseev, M. C. Ziskin, N. V Kochetkova, and M. A. Bolshakov, "Millimeter waves thermally alter the firing rate of the Lymnaea pacemaker neuron.," *Bioelectromagnetics*, vol. 18, no. 2, pp. 89–98, 1997.
[5] S. I. Alekseev and M. C. Ziskin, "Millimeter microwave effect on ion transport across lipid bilayer membranes," *Bioelectromagnetics*, 1995.
[6] A. Bellossi, G. Dubost, J. P. Moulinoux, M. Himdi, M. Ruelloux, and C. Rocher, "Biological Effects of Millimeter-Wave Irradiation on Mice—Preliminary Results," *IEEE Trans. Microw. Theory Tech.*, vol. 48, no. 11, 2000.
[7] A. Beneduci, G. Chidichimo, R. De Rose, L. Filippelli, S. V Straface, and S. Venuta, "Frequency and irradiation time-dependant antiproliferative effect of low-power millimeter waves on RPMI 7932 human melanoma cell line.," *Anticancer Res.*, vol. 25, no. 2A, pp. 1023–8, 2005.
[8] A. Beneduci, G. Chidichimo, S. Tripepi, and E. Perrotta, "Transmission electron microscopy study of the effects produced by wide-band low-power millimeter waves on MCF-7 human breast cancer cells in culture.," *Anticancer Res.*, vol. 25, no. 2A, pp. 1009–13, 2005.
[9] A. Beneduci, G. Chidichimo, S. Tripepi, E. Perrotta, and F. Cufone, "Antiproliferative effect of millimeter radiation on human erythromyeloid leukemia cell line K562 in culture: Ultrastructural- and metabolic-induced changes," *Bioelectrochemistry*, vol. 70, no. 2, pp. 214–220, May 2007.
[10] A. Beneduci, "Evaluation of the Potential In Vitro Antiproliferative Effects of Millimeter Waves at Some Therapeutic Frequencies on RPMI 7932 Human Skin Malignant Melanoma Cells," *Cell Biochem. Biophys.*, vol. 55, no. 1, pp. 25–32, Sep. 2009.
[11] L. G. Bush, D. W. Hill, A. Riazi, L. J. Stensaas, L. M. Partlow, and O. P. Gandhi, "Effects of millimeter-wave radiation on monolayer cell cultures. III. A search for frequency-specific athermal biological effects on protein synthesis," *Bioelectromagnetics*, 1981.
[12] I. Chatterjee, J. Yoon, and R. Wiese, "Millimeter wave bioeffects at 94 GHz on skeletal muscle contraction," *2013 IEEE Top.*, pp. 67–69, 2013.
[13] Q. Chen, Q. L. Zeng, D. Q. Lu, and H. Chiang, "Millimeter Wave Exposure Reverses TPA Suppression of Gap Junction Intercellular Communication in HaCaT Human Keratinocytes," *Bioelectromagnetics*, vol. 25, no. 1, pp. 1–4, 2004.
[14] S. D'Agostino *et al.*, "Extremely High Frequency Electromagnetic Fields Facilitate Electrical Signal Propagation by Increasing Transmembrane Potassium Efflux in an Artificial Axon Model," *Sci. Rep.*, 2018.
[15] A. Deghoyan, A. Heqimyan, A. Nikoghosyan, E. Dadasyan, and S. Ayrapetyan, "Cell bathing medium as a target for non thermal effect of millimeter waves.," *Electromagn. Biol. Med.*, vol. 31, no. 2, pp. 132–42, 2012.
[16] S. J.-P. Egot-Lemaire and M. C. Ziskin, "Dielectric properties of human skin at an acupuncture point in the 50-75 GHz frequency range: a pilot study.," *Bioelectromagnetics*, vol. 32, no. 5, pp. 360–6, Jul. 2011.
[17] V. Franchini *et al.*, "Genotoxic Effects in Human Fibroblasts Exposed to Microwave Radiation.," *Health Phys.*, vol. 115, no. 1, pp. 126–139, Jul. 2018.
[18] M. R. Frei, K. L. Ryan, R. E. Berger, and J. R. Jauchem, "Sustained 35-GHz radiofrequency irradiation induces circulatory failure.," *Shock*, vol. 4, no. 4, pp. 289–93, Oct. 1995.
[19] A. B. Gapeyev, T. P. Kulagina, A. V. Aripovsky, and N. K. Chemeris, "The role of fatty acids in anti-inflammatory effects of low-intensity extremely high-frequency electromagnetic radiation," *Bioelectromagnetics*, vol. 32, no. 5, pp. 388–395, 2011.
[20] A. B. Gapeyev, A. B. Gapeyev, E. N. Mikhailik, E. N. Mikhailik, N. K. Chemeris, and N. K. Chemeris, "Anti-inflammatory effects of low-intensity

extremely high-frequency electromagnetic radiation: frequency and power dependence.," *Bioelectromagnetics*, vol. 29, no. 3, pp. 197–206, 2008.
[21] A. B. Gapeyev, V. G. Safronova, N. K. Chemeris, and E. E. Fesenko, "Inhibition of the production of reactive oxygen species in mouse peritoneal neutrophils by millimeter wave radiation in the near and far field zones of the radiator," *Bioelectrochemistry Bioenerg.*, vol. 43, no. 2, pp. 217–220, Aug. 1997.
[22] V. Geletyuk and V. Kazachenko, "Dual effects of microwaves on single Ca 2+-activated K+ channels in cultured kidney cells Vero," *FEBS Lett.*, vol. 359, no. 1, pp. 85–88, 1995.
[23] W. Grundier and F. Keilmann, "Nonthermal Effects of Millimeter Microwaves on Yeast Growth," *Zeitschrift für Naturforsch. C*, vol. 33, no. 1–2, pp. 15–22, Feb. 1978.
[24] A. J. Haas, Y. Le Page, M. Zhadobov, A. Boriskin, R. Sauleau, and Y. Le Dréan, "Impact of 60-GHz millimeter waves on stress and pain-related protein expression in differentiating neuron-like cells," *Bioelectromagnetics*, vol. 37, no. 7, pp. 444–454, Oct. 2016.
[25] A. J. Haas, Y. Le Page, M. Zhadobov, R. Sauleau, Y. Le Dréan, and C. Saligaut, "Effect of acute millimeter wave exposure on dopamine metabolism of NGF-treated PC12 cells.," *J. Radiat. Res.*, vol. 58, no. 4, pp. 439–445, Jul. 2017.
[26] A. J. Haas, Y. Le Page, M. Zhadobov, R. Sauleau, and Y. Le Dréan, "Effects of 60-GHz millimeter waves on neurite outgrowth in PC12 cells using high-content screening," *Neurosci. Lett.*, vol. 618, pp. 58–65, Apr. 2016.
[27] D. Habauzit *et al.*, "Transcriptome analysis reveals the contribution of thermal and the specific effects in cellular response to millimeter wave exposure," *PLoS One*, vol. 9, no. 10, 2014.
[28] V. B. Ivanov, T. I. Subbotina, A. A. Khadartsev, M. A. Yashin, and A. A. Yashin, "Exposure to low-intensive superhigh frequency electromagnetic field as a factor of carcinogenesis in experimental animals," *Bull. Exp. Biol. Med.*, vol. 139, no. 2, pp. 241–244, 2005.
[29] J. R. Jauchem, K. L. Ryan, and T. J. Walters, "Pathophysiological alterations induced by sustained 35-GHz radio-frequency energy heating," *J. Basic Clin. Physiol. Pharmacol.*, vol. 27, no. 1, pp. 79–89, 2016.
[30] M. Kojima *et al.*, "Acute ocular injuries caused by 60-Ghz millimeter-wave exposure," *Heal. Phys*, vol. 97, no. 3, pp. 212–218, 2009.
[31] M. Kojima *et al.*, "Ocular Effects of Exposure to 40, 75, and 95 GHz Millimeter Waves," *J. Infrared, Millimeter, Terahertz Waves*, vol. 39, no. 9, pp. 912–925, Sep. 2018.
[32] A. Korenstein-Ilan, A. Barbul, P. Hasin, A. Eliran, A. Gover, and R. Korenstein, "Terahertz Radiation Increases Genomic Instability in Human Lymphocytes," *Radiat. Res.*, vol. 170, no. 2, pp. 224–234, 2008.
[33] C. Koschnitzke, F. Kremer, L. Santo, P. Quick, and A. Poglitsch, "A Non-Thermal Effect of Millimeter Wave Radiation on the Puffing of Giant Chromosomes," *Zeitschrift für Naturforsch. C*, vol. 38, no. 9–10, pp. 883–886, Oct. 1983.
[34] S. Koyama *et al.*, "Effects of long-term exposure to 60 GHz millimeter-wavelength radiation on the genotoxicity and heat shock protein (HSP) expression of cells derived from human eye," *Int. J. Environ. Res. Public Health*, vol. 13, no. 8, 2016.
[35] H. A. Kues, S. A. D. Anna, R. Osiander, W. R. Green, and J. C. Monahan, "Absence of Ocular Effects After Either Single or Repeated Exposure to 10 mW / cm 2 From a 60 GHz CW Source," *Bioelectromagnetics*, vol. 473, no. January, pp. 463–473, 1999.
[36] C. Le Quément *et al.*, "Whole-genome expression analysis in primary human keratinocyte cell cultures exposed to 60 GHz radiation," *Bioelectromagnetics*, vol. 33, no. 2, pp. 147–158, Feb. 2012.
[37] C. LeQuément, C. N. Nicolaz, D. Habauzit, M. Zhadobov, R. Sauleau, and Y. Le Dréan, "Impact of 60-GHz millimeter waves and corresponding heat

effect on endoplasmic reticulum stress sensor gene expression," *Bioelectromagnetics*, vol. 35, no. 6, pp. 444–451, 2014.
[38] X. Li *et al.*, "Millimeter wave treatment promotes chondrocyte proliferation via G 1/S cell cycle transition," *Int. J. Mol. Med.*, vol. 29, no. 5, pp. 823–831, 2012.
[39] M. K. Logani, A. Agelan, and M. C. Ziskin, "EFFECT OF MILLIMETER WAVE RADIATION ON CATALASE ACTIVITY," *Electromagn. Biol. Med.*, vol. 21, no. 3, pp. 303–308, Jan. 2002.
[40] M. K. Logani, A. Anga, I. Szabo, A. Agelan, A. R. Irizarry, and M. C. Ziskin, "Effect of millimeter waves on cyclophosphamide induced suppression of the immune system.," *Bioelectromagnetics*, vol. 23, no. 8, pp. 614–621, Dec. 2002.
[41] V. Makar, M. Logani, I. Szabo, and M. Zlskin, "Effect of Millimeter Waves on Cyclophosphamide Induced Suppression of T Cell Functions," *Bioelectromagnetics*, vol. 24, no. 5, pp. 356–365, 2003.
[42] V. R. Makar, M. K. Logani, A. Bhanushali, S. I. Alekseev, and M. C. Ziskin, "Effect of cyclophosphamide and 61.22 GHz millimeter waves on T-cell, B-cell, and macrophage functions," *Bioelectromagnetics*, vol. 27, no. 6, pp. 458–466, 2006.
[43] V. R. Makar, M. K. Logani, A. Bhanushali, M. Kataoka, and M. C. Ziskin, "Effect of millimeter waves on natural killer cell activation," *Bioelectromagnetics*, 2005.
[44] P. A. Mason *et al.*, "Lack of effect of 94 GHz radio frequency radiation exposure in an animal model of skin carcinogenesis in the radio frequency radiation (RFR) band is mutagenic , as either a promoter or co-promoter in some animal models of carcinogenesis . Recent develop," *Carcinogenesis*, vol. 22, no. 10, pp. 1701–1708, 2001.
[45] N. J. Millenbaugh *et al.*, "Comparison of blood pressure and thermal responses in rats exposed to millimeter wave energy or environmental heat," *Shock*, vol. 25, no. 6, pp. 625–632, 2006.
[46] N. J. Millenbaugh *et al.*, "Gene expression changes in the skin of rats induced by prolonged 35 GHz millimeter-wave exposure.," *Radiat. Res.*, vol. 169, no. 3, pp. 288–300, 2008.
[47] L. Narinyan and S. Ayrapetyan, "Cyclic AMP-dependent signaling system is a primary metabolic target for non-thermal effect of microwaves on heart muscle hydration," *Electromagn. Biol. Med.*, vol. 36, no. 2, pp. 182–191, Apr. 2017.
[48] T. H. P. Nguyen *et al.*, "The bioeffects resulting from prokaryotic cells and yeast being exposed to an 18 GHz electromagnetic field," *PLoS One*, 2016.
[49] C. N. Nicolaz *et al.*, "Study of narrow band millimeter-wave potential interactions with endoplasmic reticulum stress sensor genes," *Bioelectromagnetics*, vol. 30, no. 5, pp. 365–373, 2009.
[50] N. C. Nicolaz *et al.*, "Absence of direct effect of low-power millimeter-wave radiation at 60.4 GHz on endoplasmic reticulum stress.," *Cell Biol. Toxicol.*, vol. 25, no. 5, pp. 471–8, Oct. 2009.
[51] A. G. Pakhomov, H. K. Prol, S. P. Mathur, Y. Akyel, and C. B. G. B. G. Campbell, "Role of field intensity in the biological effectiveness of millimeter waves at a resonance frequency," *Bioelectrochemistry Bioenerg.*, vol. 43, no. 1, pp. 27–33, Jun. 1997.
[52] A. G. Pakhomov, H. K. Prol, S. P. Mathur, Y. Akyel, and C. B. Campbell, "Search for frequency-specific effects of millimeter-wave radiation on isolated nerve function.," *Bioelectromagnetics*, vol. 18, no. 4, pp. 324–334, 1997.
[53] L. M. Partlow, L. G. Bush, L. J. Stensaas, D. W. Hill, A. Riazi, and O. P. Gandhi, "Effects of millimeter-wave radiation on monolayer cell cultures. I. Design and validation of a novel exposure system," *Bioelectromagnetics*, 1981.

[54] T. Partyla, H. Hacker, H. Edinger, B. Leutzow, J. Lange, and T. Usichenko, "Remote Effects of Electromagnetic Millimeter Waves on Experimentally Induced Cold Pain: A Double-Blinded Crossover Investigation in Healthy Volunteers," *Anesth. Analg.*, vol. 124, no. 3, pp. 980–985, 2017.
[55] V. Pikov, X. Arakaki, M. Harrington, S. E. Fraser, and P. H. Siegel, "Modulation of neuronal activity and plasma membrane properties with low-power millimeter waves in organotypic cortical slices," *J. Neural Eng.*, vol. 7, no. 4, p. 045003, Aug. 2010.
[56] A. Radzievsky, O. Gordiienko, A. Cowan, S. . Alekseev, and M. . Ziskin, "Millimeter-Wave-Induced Hypoalgesia in Mice: Dependence on Type of Experimental Pain," *IEEE Trans. Plasma Sci.*, vol. 32, no. 4, pp. 1634–1643, Aug. 2004.
[57] A. A. Radzievsky, A. Cowan, C. Byrd, A. A. Radzievsky, and M. C. Ziskin, "Single millimeter wave treatment does not impair gastrointestinal transit in mice," *Life Sci.*, vol. 71, no. 15, pp. 1763–1770, 2002.
[58] A. A. Radzievsky, O. V. Gordiienko, S. Alekseev, I. Szabo, A. Cowan, and M. C. Ziskin, "Electromagnetic millimeter wave induced hypoalgesia: Frequency dependence and involvement of endogenous opioids," *Bioelectromagnetics*, vol. 29, no. 4, pp. 284–295, May 2008.
[59] A. Radzievsky, O. Gordiienko, I. Szabo, S. I. Alekseev, and M. C. Ziskin, "Millimeter wave-induced suppression of B16 F10 melanoma growth in mice: involvement of endogenous opioids.," *Bioelectromagnetics*, vol. 25, no. 6, pp. 466–73, Sep. 2004.
[60] A. A. Radzievsky, M. A. Rojavin, A. Cowan, S. I. Alekseev, A. A. Radzievsky, and M. C. Ziskin, "Peripheral neural system involvement in hypoalgesic effect of electromagnetic millimeter waves," *Life Sci.*, vol. 68, no. 10, pp. 1143–1151, 2001.
[61] A. A. Radzievsky, M. A. Rojavin, A. Cowan, S. I. Alekseev, and M. C. Ziskin, "Hypoalgesic effect of millimeter waves in mice: Dependence on the site of exposure," *Life Sci.*, vol. 66, no. 21, pp. 2101–2111, 2000.
[62] A. A. Radzievsky, M. A. Rojavin, A. Cowan, and M. C. Ziskin, "Suppression of pain sensation caused by millimeter waves: a double-blinded, cross-over, prospective human volunteer study.," *Anesth. Analg.*, vol. 88, no. 4, pp. 836–40, Apr. 1999.
[63] M. A. Rojavin, A. Cowan, A. A. Radzievsky, and M. C. Ziskin, "Antipruritic effect of millimeter waves in mice: evidence for opioid involvement," *Life Sci.*, vol. 63, no. 18, pp. 251–257, 1998.
[64] M. A. Rojavin, A. A. Radzievsky, A. Cowan, and M. C. Ziskin, "Pain relief caused by millimeter waves in mice: results of cold water tail flick tests," *Int J Radiat Biol*, vol. 76, no. 4, pp. 575–579, 2000.
[65] S. Romanenko, P. H. Siegel, D. A. Wagenaar, and V. Pikov, "Effects of millimeter wave irradiation and equivalent thermal heating on the activity of individual neurons in the leech ganglion.," *J. Neurophysiol.*, vol. 112, no. 10, pp. 2423–31, 2014.
[66] K. L. Ryan, M. R. Frei, R. E. Berger, and J. R. Jauchem, "Does nitric oxide mediate circulatory failure induced by 35-GHz microwave heating?," *Shock*, vol. 6, no. 1, pp. 71–6, Jul. 1996.
[67] K. L. Ryan, M. R. Frei, and J. R. Jauchem, "Circulatory failure induced by 35 GHz microwave heating: effects of chronic nitric oxide synthesis inhibition.," *Shock*, vol. 7, no. 1, pp. 70–6, Jan. 1997.
[68] V. G. Safronova, A. G. Gabdoulkhakova, and B. F. Santalov, "Immunomodulating Action of Low Intensity Millimeter Waves on Primed Neutrophils," *Bioelectromagnetics*, vol. 23, no. 8, pp. 599–606, 2002.
[69] V. O. Samoilov, E. B. Shadrin, E. B. Filippova, Y. Katsnelson, H. Backhoff, and M. Eventov, "The effect of transcranial electromagnetic brain stimulation on the acquisition of the conditioned response in rats," *Biophysics (Oxf).*, vol. 60, no. 2, pp. 303–308, Mar. 2015.
[70] A. Samsonov and S. V Popov, "The effect of a 94 GHz electromagnetic field on neuronal microtubules.," *Bioelectromagnetics*, vol. 34, no. 2, pp. 133–44, 2013.

[71] S. N. Shanin, E. G. Rybakina, N. N. Novikova, I. A. Kozinets, V. J. Rogers, and E. A. Korneva, "Natural killer cell cytotoxic activity and c-Fos protein synthesis in rat hypothalamic cells after painful electric stimulation of the hind limbs and EHF irradiation of the skin," *Med Sci Monit*, vol. 11, no. 9, pp. BR309-15, 2005.

[72] M. G. Shapiro, M. F. Priest, P. H. Siegel, and F. Bezanilla, "Thermal Mechanisms of Millimeter Wave Stimulation of Excitable Cells," *Biophys. J.*, vol. 104, no. 12, pp. 2622–2628, Jun. 2013.

[73] Y. G. Shckorbatov, N. N. Grigoryeva, V. G. Shakhbazov, V. A. Grabina, and A. M. Bogoslavsky, "Microwave irradiation influences on the state of human cell nuclei.," *Bioelectromagnetics*, vol. 19, no. May 1999, pp. 414–419, 1998.

[74] I. B. Sivachenko, D. S. Medvedev, I. D. Molodtsova, S. S. Panteleev, A. Y. Sokolov, and O. A. Lyubashina, "Effects of Millimeter-Wave Electromagnetic Radiation on the Experimental Model of Migraine," *Bull. Exp. Biol. Med.*, vol. 160, no. 4, pp. 425–428, 2016.

[75] Y. Soubere Mahamoud *et al.*, "Additive Effects of Millimeter Waves and 2-Deoxyglucose Co-Exposure on the Human Keratinocyte Transcriptome," *PLoS One*, vol. 11, no. 8, p. e0160810, Aug. 2016.

[76] L. J. Stensaas *et al.*, "Effects of millimeter-wave radiation on monolayer cell cultures. II. Scanning and transmission electron microscopy," *Bioelectromagnetics*, 1981.

[77] S. Sun, I. Titushkin, J. Varner, and M. Cho, "Millimeter Wave-induced Modulation of Calcium Dynamics in an Engineered Skin Co-culture Model: Role of Secreted ATP on Calcium Spiking," *J. Radiat. Res.*, vol. 53, no. 2, pp. 159–167, 2012.

[78] R. K. Sypniewska *et al.*, "Protein changes in macrophages induced by plasma from rats exposed to 35 GHz millimeter waves.," *Bioelectromagnetics*, vol. 31, no. 8, pp. 656–63, Dec. 2010.

[79] I. Szabo, J. Kappelmayer, S. I. Alekseev, and M. C. Ziskin, "Millimeter wave induced reversible externalization of phosphatidylserine molecules in cells exposed in vitro," *Bioelectromagnetics*, vol. 27, no. 3, pp. 233–244, 2006.

[80] I. Szabo, M. R. Manning, A. A. Radzlevsky, M. A. Wetzel, T. J. Rogers, and M. C. Ziskin, "Low Power Millimeter Wave Irradiation Exerts No Harmful Effect on Human Keratinocytes In Vitro," *Bioelectromagnetics*, vol. 24, no. 3, pp. 165–173, 2003.

[81] I. Szabo, M. A. Rojavin, T. J. Rogers, and M. C. Ziskin, "Reactions of keratinocytes to in vitro millimeter wave exposure," *Bioelectromagnetics*, vol. 22, no. 5, pp. 358–364, 2001.

[82] I. A. Titushkin, V. S. Rao, W. F. Pickard, E. G. Moros, G. Shafirstein, and M. R. Cho, "Altered Calcium Dynamics Mediates P19-Derived Neuron-Like Cell Responses to Millimeter-Wave Radiation," *Radiat. Res.*, vol. 172, no. 6, pp. 725–736, 2009.

[83] Y. Tong *et al.*, "Millimeter-wave exposure promotes the differentiation of bone marrow stromal cells into cells with a neural phenotype.," *J. Huazhong Univ. Sci. Technolog. Med. Sci.*, vol. 29, no. 4, pp. 409–12, 2009.

[84] Vijayalaxmi, M. K. Logani, A. Bhanushali, M. C. Ziskin, and T. J. Prihoda, "Micronuclei in peripheral blood and bone marrow cells of mice exposed to 42 GHz electromagnetic millimeter waves.," *Radiat. Res.*, vol. 161, no. 3, pp. 341–5, Mar. 2004.

[85] I. I. Vlasova, E. V Mikhalchik, A. A. Gusev, N. G. Balabushevich, S. A. Gusev, and K. D. Kazarinov, "Extremely high-frequency electromagnetic radiation enhances neutrophil response to particulate agonists," *Bioelectromagnetics*, vol. 39, no. 2, pp. 144–155, Feb. 2018.

[86] N. A. Volkova, E. V Pavlovich, A. A. Gapon, and O. T. Nikolov, "Effects of Millimeter-Wave Electromagnetic Exposure on the Morphology and Function of Human Cryopreserved Spermatozoa," *Bull. Exp. Biol. Med.*, vol. 157, no. 5, pp. 574–576, Sep. 2014.

[87] G. Wu *et al.*, “Millimeter wave treatment inhibits the mitochondrion-dependent apoptosis pathway in chondrocytes,” *Mol. Med. Rep.*, vol. 4, no. 5, pp. 1001–1006, 2011.

[88] G. W. Wu *et al.*, “Experimental study of millimeter wave-induced differentiation of bone marrow mesenchymal stem cells into chondrocytes,” *Int J Mol Med*, vol. 23, no. 4, pp. 461–467, 2009.

[89] L. Xia, Q.-L. Luo, H.-D. Lin, J.-L. Zhang, H. Guo, and C.-Q. He, “The effect of different treatment time of millimeter wave on chondrocyte apoptosis, caspase-3, caspase-8, and MMP-13 expression in rabbit surgically induced model of knee osteoarthritis,” *Rheumatol. Int.*, vol. 32, no. 9, pp. 2847–2856, Sep. 2012.

[90] T. Xie *et al.*, “EEG CHANGES AS HEAT STRESS REACTIONS IN RATS IRRADIATED BY HIGH INTENSITY 35 GHz MILLIMETER WAVES,” *Health Phys.*, vol. 100, no. 6, pp. 632–640, Jun. 2011.

[91] N. Yaekashiwa, S. Otsuki, S. Hayashi, and K. Kawase, “Investigation of the non-thermal effects of exposing cells to 70-300 GHz irradiation using a widely tunable source.,” *J. Radiat. Res.*, vol. 59, no. 2, pp. 116–121, Mar. 2018.

[92] G. Yu *et al.*, “A study on biological effects of low-intensity millimeter waves,” *IEEE Trans. Plasma Sci.*, vol. 30, no. 4, pp. 1489–1496, 2002.

[93] M. Zhadobov, F. Desmots, D. Thouroude, D. Michel, and Y. Drean, “Evaluation of the potential biological effects of the 60-GHz millimeter waves upon human cells,” *IEEE Trans. Antennas Propag.*, vol. 57, no. 10, pp. 2949–2956, 2009.

[94] M. Zhadobov *et al.*, “Low-power millimeter wave radiations do not alter stress-sensitive gene expression of chaperone proteins,” *Bioelectromagnetics*, vol. 28, no. 3, pp. 188–196, 2007.
